# Agricultural management and plant selection interactively affect rhizosphere microbial community structure and nitrogen cycling

**DOI:** 10.1186/s40168-019-0756-9

**Published:** 2019-11-07

**Authors:** Jennifer E. Schmidt, Angela D. Kent, Vanessa L. Brisson, Amélie C. M. Gaudin

**Affiliations:** 1 Department of Plant Sciences, University of California, Davis, One Shields Avenue, Davis, CA 95616 USA; 2 Department of Natural Resources and Environmental Sciences, University of Illinois at Urbana-Champaign, N-215 Turner Hall, MC-047, 1102 S. Goodwin Avenue, Urbana, IL USA; 3 Lawrence Berkeley National Laboratory, 1 Cyclotron Road, Berkeley, CA 94720 USA; 4 The DOE Joint Genome Institute, 2800 Mitchell Drive, Walnut Creek, CA 94598 USA

**Keywords:** Rhizosphere, Agricultural management, Soil microbial community, Nitrogen cycling, Quantitative PCR, Agroecosystem, Adaptive feedbacks

## Abstract

**Background:**

Rhizosphere microbial communities are key regulators of plant performance, yet few studies have assessed the impact of different management approaches on the rhizosphere microbiomes of major crops. Rhizosphere microbial communities are shaped by interactions between agricultural management and host selection processes, but studies often consider these factors individually rather than in combination. We tested the impacts of management (M) and rhizosphere effects (R) on microbial community structure and co-occurrence networks of maize roots collected from long-term conventionally and organically managed maize-tomato agroecosystems. We also explored the interaction between these factors (M × R) and how it impacts rhizosphere microbial diversity and composition, differential abundance, indicator taxa, co-occurrence network structure, and microbial nitrogen-cycling processes.

**Results:**

Host selection processes moderate the influence of agricultural management on rhizosphere microbial communities, although bacteria and fungi respond differently to plant selection and agricultural management. We found that plants recruit management-system-specific taxa and shift N-cycling pathways in the rhizosphere, distinguishing this soil compartment from bulk soil. Rhizosphere microbiomes from conventional and organic systems were more similar in diversity and network structure than communities from their respective bulk soils, and community composition was affected by both M and R effects. In contrast, fungal community composition was affected only by management, and network structure only by plant selection. Quantification of six nitrogen-cycling genes (*nifH*, *amoA* [bacterial and archaeal], *nirK*, *nrfA*, and *nosZ*) revealed that only *nosZ* abundance was affected by management and was higher in the organic system.

**Conclusions:**

Plant selection interacts with conventional and organic management practices to shape rhizosphere microbial community composition, co-occurrence patterns, and at least one nitrogen-cycling process. Reframing research priorities to better understand adaptive plant-microbe feedbacks and include roots as a significant moderating influence of management outcomes could help guide plant-oriented strategies to improve productivity and agroecosystem sustainability.

## Background

Soil microbial communities are shaped by diverse, interacting forces. In agroecosystems, management practices such as crop rotation, fertilization, and tillage alter soil physicochemical parameters, influencing the diversity and composition of bulk soil bacterial and fungal communities [1]. Plant roots create additional complexity, establishing resource-rich hotspots with distinct properties from the bulk soil and selectively recruiting microbial communities in the rhizosphere [2, 3]. Root uptake of ions and water coupled with exudation of carbon-rich compounds results in a rhizosphere soil compartment where microbial cycling of nitrogen, phosphorous, and other nutrients is rapid, dynamic, and competitive in comparison to the bulk soil. Although impacts of agricultural management and the rhizosphere environment on microbiomes and their ecological outcomes have frequently been analyzed separately, understanding interactions has important implications for assembly, ecology, and functioning of rhizosphere microbial communities which are critical to plant health and productivity [4].

Agricultural management establishes soil physicochemical properties that influence microbial community composition, structure, and nutrient-cycling functions. Organic fertilizer increases bulk soil microbial diversity and heterogeneity [5], and organically managed systems differ from conventional systems in bacterial and fungal community composition [1, 6–8]. Co-occurrence network analysis has shown that these taxonomic shifts can shape patterns of ecological interactions regulating structure, function, and potential resilience of soil microbial communities [9–12]. In fact, nutrient management strategies are strong drivers of co-occurrence network structural properties, although outcomes across regions and agroecosystems are inconsistent and also a function of other environmental and management factors [13–15].

Plant roots are similarly powerful drivers of microbial community assembly, creating rhizosphere communities that are taxonomically and functionally distinct from bulk soil [16]. The strength of plant selection, or rhizosphere effect, is evident in observations of core microbiomes across different field environments [17, 18]. As for management, plant effects on microbial communities also extend beyond taxonomy to network structure. Rhizosphere networks have frequently been found to be smaller, less densely connected, and less complex than bulk soil networks [3, 19–21], although counterexamples exist [22]. Whether plasticity in rhizosphere recruitment can occur across management gradients and how such plasticity could impact plant adaptation to varying resource availabilities in agroecosystems remains unclear.

The potential for adaptive plant-microbe feedbacks is especially relevant for acquisition of nitrogen (N), an essential nutrient whose availability in agroecosystems is controlled by interactions between fertility management practices and microbial metabolic processes. Microbial communities supply plant-available N through biological N fixation and mineralization of organic forms, and limit N losses by immobilizing it in soil organic matter. Conventional and organic agroecosystems establish unique contexts in which these transformations occur, shaping microbial communities through system-specific differences in soil N availability and dominant N forms [23–26] as well as quantity and quality of soil organic matter [27]. Organic fertility inputs such as compost and cover crop residues alter the abundance, diversity, and activity of various nitrogen-cycling microorganisms [7, 28–30], while synthetic fertilizers mainly increase the abundance of Acidobacteria [1] and can decrease the abundance of ammonia-oxidizing archaea [31]. Synthetic fertilizers may affect microbial community structure via changes in pH, increasing the abundance of acid-tolerant taxa indirectly through soil acidification, or may alter the relative abundance of specific taxa even when pH is relatively constant [32]. Changes in microbial community structure and activity in bulk soil affect not just the rates but also the outcomes of agriculturally and environmentally relevant N-cycling processes such as denitrification [27]. Roots are also key regulators of N transformations, leading to higher rates of N cycling that are more closely coupled to plant demand in the rhizosphere than in bulk soil compartments [33]. The maize rhizosphere harbors a distinct denitrifier community [34] and is enriched in functional genes related to nitrogen fixation (*nifH*), ammonification (*gdh*, *ureC*), nitrification (*amoA*, *hao*), and denitrification (*narG*, *nirS*/*nirK*, *norB*, *nosZ*) relative to soil beyond the influence of roots [35–37]. Understanding regulation of tight coupling of rhizosphere N cycling processes to plant demand [38] could provide new avenues for more efficient and sustainable N management, particularly in an era of global change [39].

However, it is necessary to go beyond exploration of individual effects of plant selection and agricultural management on rhizosphere microbial communities and consider how these factors interact. This knowledge can contribute to managing rhizosphere interactions that promote both plant productivity and agroecosystem sustainability. While management-induced shifts in bulk soil microbiomes affect environmental outcomes, plant-regulated rhizosphere communities are more directly relevant to yield outcomes. Improved understanding of how plant selection changes across management systems is thus an essential component of sustainable intensification strategies that decouple agroecosystem productivity from environmental footprints, particularly in organic systems where yields are formed through transformation of natural resources rather than transformation of external synthetic inputs [40].

**Fig. 1:**
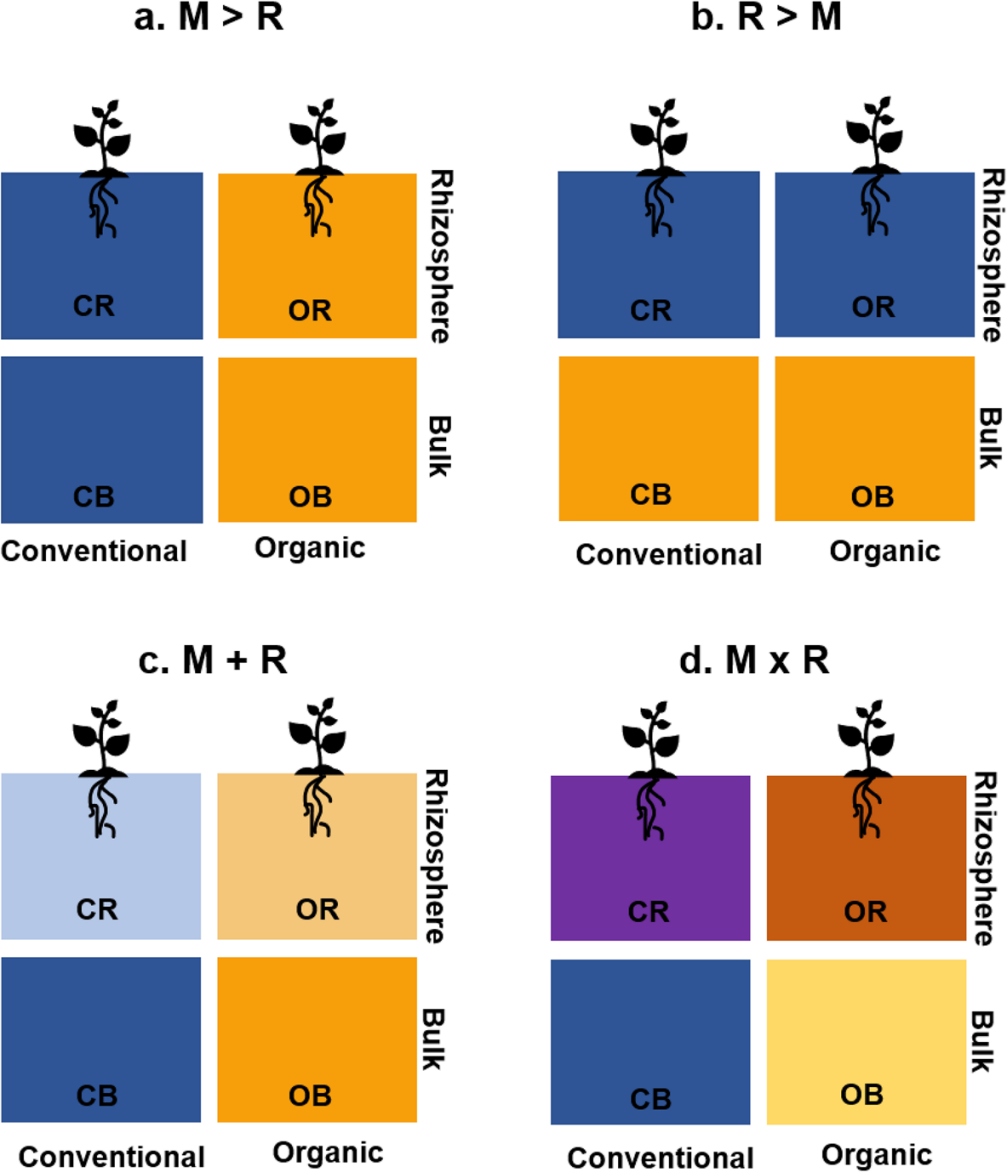
Potential relationships between management and rhizosphere effects. Conceptual framework of scenarios for management (M) and rhizosphere (R) effects on microbiomes. **a** M effects could be stronger than R, leading to stronger differences between microbial communities in different systems than between bulk soil and rhizosphere communities. **b** Conversely, R effects could be stronger than M, leading to distinct bulk and rhizosphere communities across management systems. **c** If M and R effects are additive, plant effects act consistently on distinct bulk soil pools. Rhizosphere communities thus differ from bulk soil and differ from one another by the same amount as their respective bulk soil communities do. **d** An M × R interaction is present, and the magnitude or direction of the rhizosphere effect could differ between systems. In addition, differences between rhizosphere communities could be greater than differences between bulk soil communities

When management (M) and plant rhizosphere (R) effects shape rhizosphere microbial communities, a number of scenarios are possible: one could be greater than the other (M > R or R > M), their effects could be additive (M + R), or they could interact (M × R) (Fig. 1). Typically, these effects are considered additive (M + R), where management shapes bulk soil communities and plant effects act consistently, such that rhizosphere communities are distinct from bulk soil and differ from one another to the same degree as their respective bulk soil communities. However, variation in rhizosphere microbiomes [30, 41–43] and co-occurrence networks [43] between management systems and the unique responses of bulk soil and rhizosphere bacteria to cropping systems [44] point toward M × R interactions shaping microbial community composition. Nonetheless, the functional significance of these interactive effects on critical functions such as N cycling is complex and remains difficult to predict. For example, biological N fixation is driven in large part by plant demand, but high inputs of synthetic fertilizer reduce rates of biological N fixation, diminishing the role of soil microbial communities in supplying plant nutrients and increasing the potential for reactive N losses [45]. Understanding how the M × R interaction affects ecological functions is thus a knowledge gap of critical agricultural and environmental relevance.

Adaptive plant-microbe feedbacks in the rhizosphere have been described for natural ecosystems [46], but whether this can occur in intensively managed agricultural systems where resources are more abundant is less clear [47]. We asked whether adaptation to contrasting management systems shifts the magnitude or direction of the rhizosphere effect on rhizosphere community composition and/or N-cycling functions across systems. For instance, can the same genotype selectively enrich adaptive functions that increase N mineralization from cover crops and compost when planted in an organic system and also reduce denitrification loss pathways from inorganic fertilizer when planted in a conventional system? We hypothesized that (a) an M × R interaction would result in differences in the magnitude or direction of the rhizosphere effect on microbial community structure and functions and that (b) differences between rhizosphere communities, co-occurrence network structure, or N-cycling processes would reflect adaptive management-system-specific shifts. To test these hypotheses, we investigated microbial community composition and co-occurrence patterns in bulk and rhizosphere samples from a single maize genotype grown in a long-term conventional-organic field trial. We further quantified the abundance of six microbial N-cycling genes as case study for M × R impacts on rhizosphere processes of agricultural relevance. Our approach integrated ordination, differential abundance and indicator species analyses, construction of co-occurrence networks, and quantitative PCR of N-cycling genes to gain a deeper understanding of the factors that shape rhizosphere community and ecological interactions.

## Results

### Microbial diversity

We observed significant rhizosphere effects on the alpha diversity of bacterial and archaeal communities (*n* = 36) at the ASV level (Additional file 8: Figure S1). At this taxonomic level, bulk soil bacterial/archaeal communities were significantly more diverse under organic management than conventional management (*p* < 0.05). However, rhizosphere bacterial/archaeal communities were equally diverse in both systems, with diversity intermediate to that of the two bulk soils. Thus, the direction of the rhizosphere effect differed between systems. For diversity and indicator species, the direction of the rhizosphere effect reflects the increase/decrease in the parameter of interest in the rhizosphere relative to bulk soil. For community composition, the direction of the rhizosphere effect was based on visualization of the vector from the bulk soil community to the rhizosphere community in multivariate ordination. While plants acted as a selective filter to decrease diversity in the rhizosphere of organically grown plants, rhizosphere bacterial/archaeal diversity was enriched in the conventional system compared to bulk soil (M × R *p* < 0.001, Additional file 8: Figure S1a). Fungal diversity did not differ between rhizosphere and bulk soil samples or between management systems at the ASV level (*n* = 36, *p* > 0.05, Additional file 8: Figure S1b).

### Microbial community composition

**Fig. 2:**
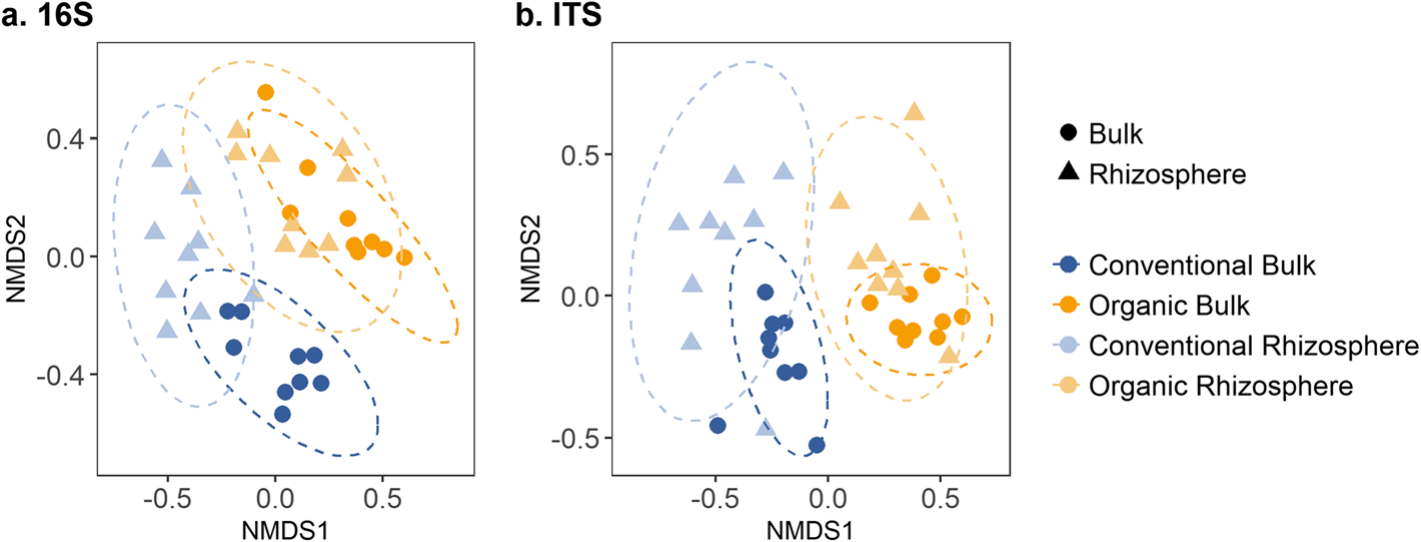
NMDS ordination of bacterial and fungal communities. **a** Bacterial communities separated by management and soil compartment (PERMANOVA *p* < 0.05). **b** Fungal communities responded to management effects but not plant influence (PERMANOVA *p* = 0.001). All ordinations were performed using ASV-level data

NMDS ordination based on Bray-Curtis distances showed that bacterial and archaeal communities were distinct between management systems and soil compartments (bulk soil or rhizosphere) at the ASV level (Fig. 2a) and all four ANOSIM pairwise comparisons were significantly different (*p* < 0.01, Additional file 3: Table S2). We observed a significant M × R interaction (*p* < 0.05), showing that the strength of plant influence on bacterial recruitment differed between management systems. We found greater differences between bulk and rhizosphere communities at the ASV level in conventional soils compared to organic (Fig. 2a, Additional file 3: Table S2).

Genus-level relative abundance data showed that Bacillus tended to be the most abundant bacterial genus, especially in CB and OR samples (Additional file 9: Figure S2a). Skermanella and Steroidobacter were also relatively common in most samples. Few differences between management systems were observed at this taxonomic level, but plant selection appeared to reduce the abundance of Pseudarthrobacter in the rhizosphere in both systems and increase the abundance of the genera RB41 and Acidibacter.

Management but not soil compartment significantly distinguished fungal communities as shown with NMDS ordination (Fig. 2b, PERMANOVA *p* = 0.001). ANOSIM pairwise comparisons supported this conclusion using a Bonferroni-adjusted *p* value of 0.0125, although the soil compartment effect in the organic system was significant at the *p* = 0.05 level (*p* = 0.04, Additional file 3: Table S2). The genera Mortierella and Cryptococcus were most abundant across all samples (Additional file 9: Figure S2b). Cystofilobasidium tended to be more abundant in the organic system, whereas members of the genera Rhizopus and Minimedusa tended to be more abundant in the conventional system. The genera Articulospora and Aspergillus appeared to respond to plant selection, with Articulospora more abundant in bulk soil and Aspergillus more abundant in the rhizosphere.

### Differentially abundant ASVs

**Fig. 3:**
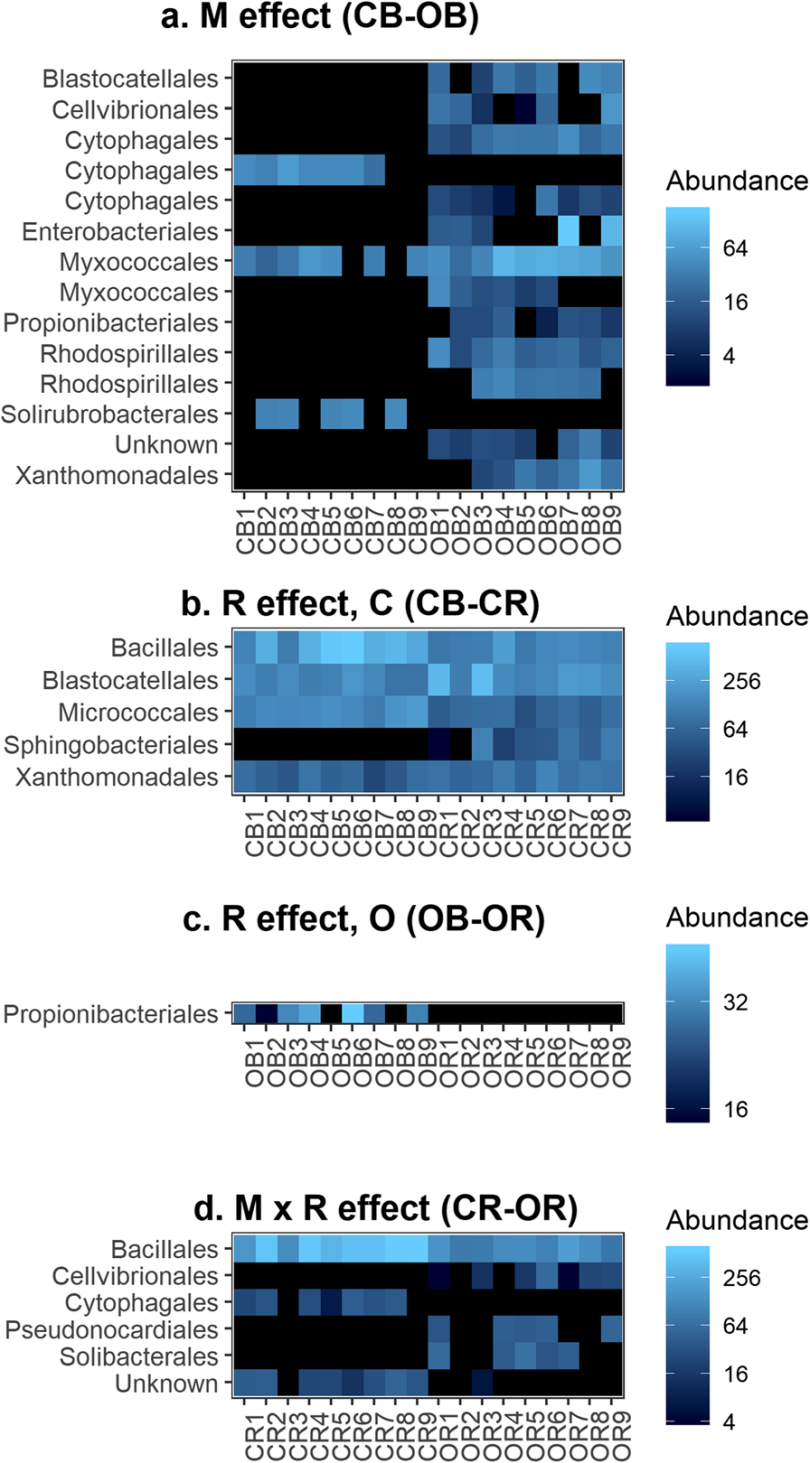
Differentially abundant bacterial ASVs. Bacterial ASVs were identified whose abundance was affected by **a**) management (M), **b**-**c**) the rhizosphere effect (R), or **d**) the M × R interaction. More bacterial ASVs differed in abundance due to management than in response to the rhizosphere effect or M × R interaction. “Unknown” indicates that the ASV was not identified at the order level. Sample names on the *x*-axis indicate the combination of management system (C conventional, O organic), soil compartment (B bulk, R rhizosphere), and replicate (plot 1 = samples 1–3, plot 2 = samples 4–6, plot 3 = samples 7–9). Only ASVs that differed significantly among treatments at the *α* = 0.0125 level are shown

**Fig. 4:**
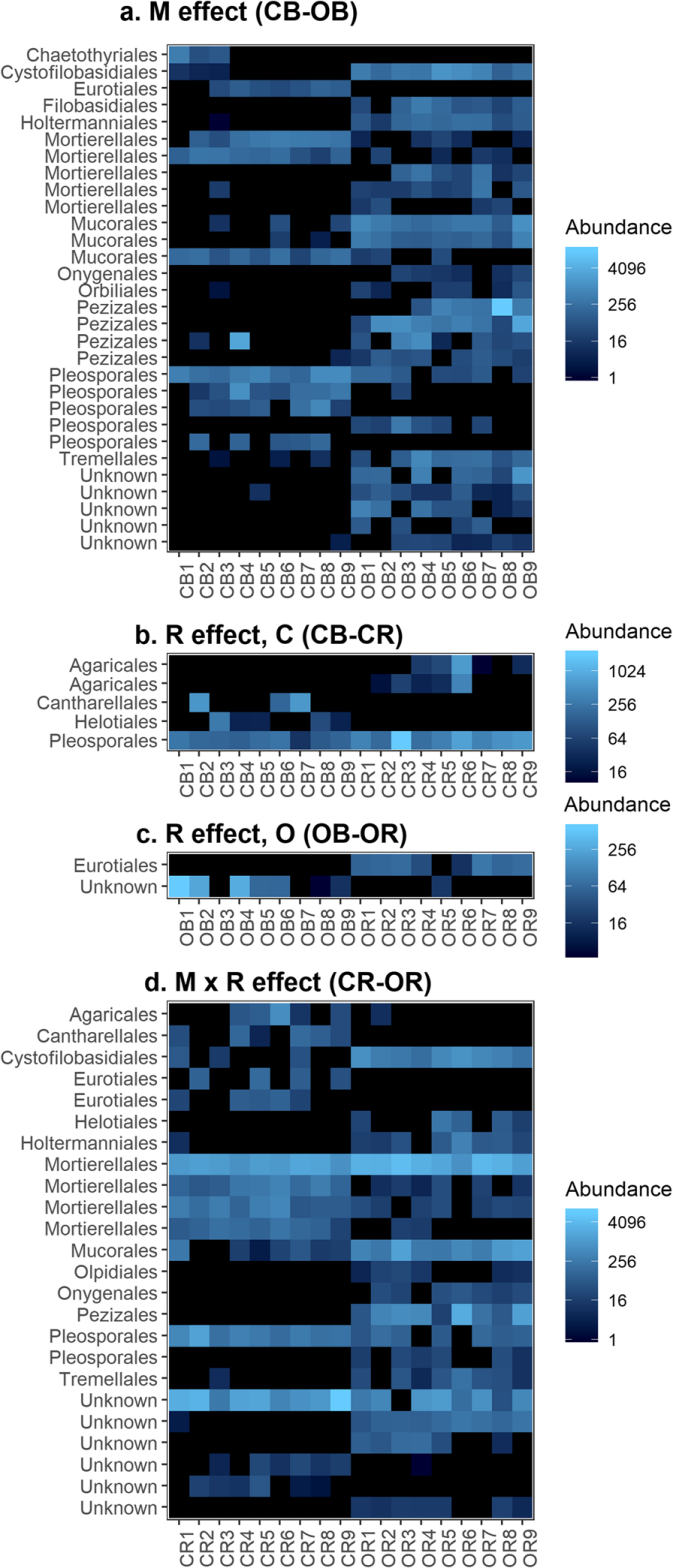
Differentially abundant fungal ASVs. Fungal ASVs were identified whose abundance was affected by **a**) management (M), **b**-**c**) the rhizosphere effect (R), or **d**) the M × R interaction. The M × R interaction was strong in fungal communities, as shown by the high number of ASVs differing in abundance between the CR and OR environments. “Unknown” indicates that the ASV was not identified at the order level. Sample names on the *x*-axis indicate the combination of management system (C conventional, O organic), soil compartment (B bulk, R rhizosphere), and replicate (plot 1 = samples 1–3, plot 2 = samples 4–6, plot 3 = samples 7–9). Only ASVs that differed significantly among treatments at the *α* = 0.0125 level are shown

Variation in community composition was investigated at greater taxonomic resolution by identifying ASVs whose abundance differed in response to management, rhizosphere effects, or their interaction (Figs. 3and 4). The greatest number of differentially abundant ASVs was observed between the organic and conventional bulk soil environments, with 14 bacterial and 30 fungal ASVs, highlighting the strong impact of management on community composition (*p* < 0.01, Figs. 3 and 4). Twelve of the 14 bacterial ASVs were more abundant in the organic system (OB), while two ASVs belonging to the orders Cytophagales and Solirubrobacterales were more abundant in conventional bulk soil (CB) (Fig. 3a). The 30 differentially abundant fungal ASVs were taxonomically diverse and 21 of 30 were more abundant in the organic system (Fig. 4a).

A greater number of ASVs showed a significant response to plant selection in conventional (CB-CR) than organic soil (OB-OR) (Fig. 3b, c and Fig. 4b, c). Five bacterial and five fungal ASVs were differentially abundant between the conventional bulk and rhizosphere soils (Figs. 3b and 4b), as compared to one bacterial and two fungal ASVs in the organic bulk and rhizosphere soils (Figs. 3c and 4c).

The number of differentially abundant taxa between the rhizosphere communities of the two systems (CR-OR) was at least as great as the number responding to within-system rhizosphere effects (Fig. 3b–d and Fig. 4b–d). More fungal than bacterial ASVs were differentially abundant between these rhizosphere communities: 24 fungal ASVs but only six bacterial ASVs were significantly different in abundance between CR and OR, indicating strong M × R interactions. The differentially abundant fungi and bacteria were evenly distributed between the two management systems. For fungi, 11 ASVs were more abundant in the rhizosphere of conventional plants and 13 were more abundant in organic. The Mortierellales were the most-represented order with four ASVs, but these were not disproportionately found in CR or OR (Additional file 9: Figure S2b).

### Indicator ASVs

A total of 74 bacterial/archaeal ASVs were identified as indicator taxa, with 27 of those specific to one environment (management system-soil compartment combination) and 47 to a combination of two environments (Additional file 10: Figure S3a, Additional file 4: Table S3). Management effects were stronger than soil compartment effects and more bacterial ASVs were management-system-specific (10 to conventional, 21 to organic) than soil-compartment-specific (5 to rhizosphere, 11 to bulk). We observed a significant M × R interaction in recruitment of unique taxa: more ASVs were unique indicators of the conventional rhizosphere communities (11 to CR vs. 5 to CB) while the opposite was true under organic management (2 to OR vs. 9 to OB). Bacterial/archaeal indicators were widely distributed phylogenetically (Additional file 4: Table S3).

Forty-nine fungal indicator ASVs were identified: 16 corresponding to one management system-soil compartment environment and 33 to two environments (Additional file 10: Figure S3b, Additional file 5: Table S4). Similar to bacterial/archaeal communities, management system had a stronger influence than the rhizosphere on indicator taxa: 12 fungal indicator ASVs were specific to conventional management, 18 to organic management, and only three to the rhizosphere and none to bulk soil.

An M × R interaction was also observed in which more fungal indicators were specific to the rhizosphere in the conventional system (9 to CR vs. 2 to CB) than in the organic system (1 to OR vs. 4 to OB).

### Network analysis

**Fig. 5:**
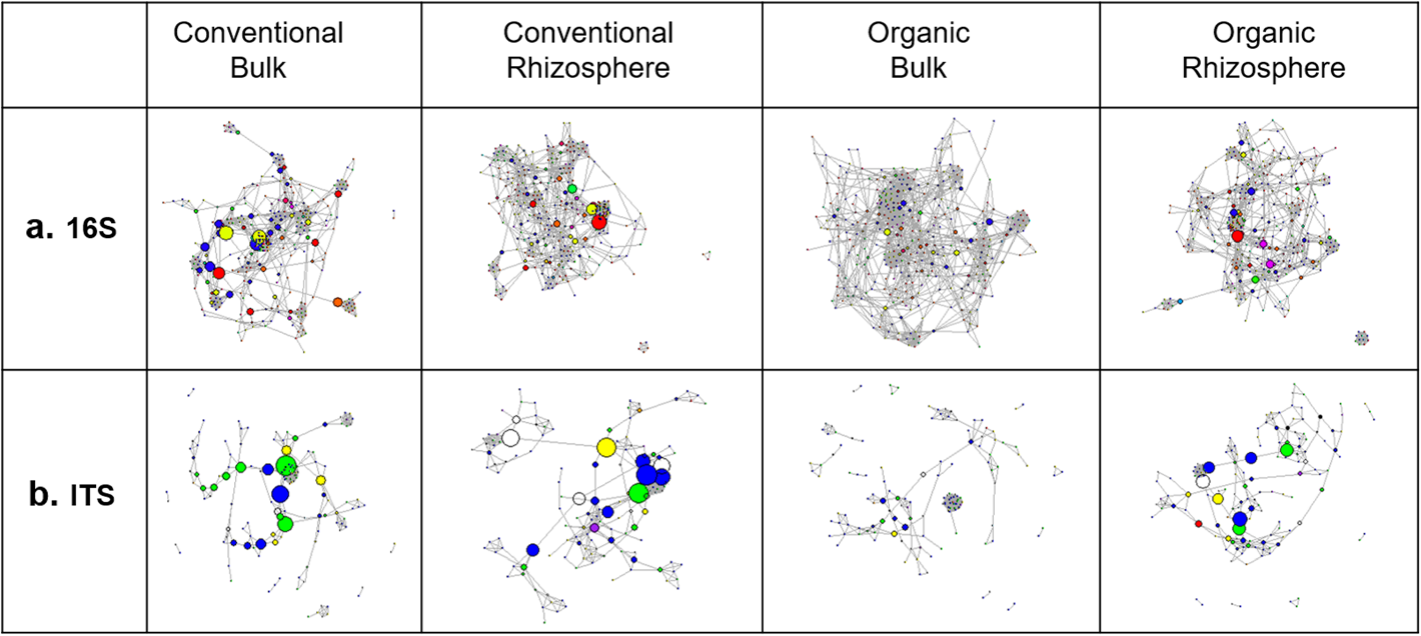
Microbial co-occurrence networks. Bacterial and fungal co-occurrence networks constructed from ASV-level data corresponding to each combination of management system and soil compartment have different structural characteristics. Nodes indicate amplicon sequence variants (ASVs) and edges represent significant co-occurrence relationships (Spearman’s *ρ* > 0.75 and *p* < 0.05). **a** For bacteria, conventional networks had more edges but the same number of nodes as compared to organic networks. Furthermore, while the rhizosphere network had fewer edges than the bulk soil network in the conventional system, the rhizosphere network had more edges than the bulk soil network in the organic system. **b** Fungal rhizosphere networks were smaller, less connected, less dense, less centralized, and more modular than the corresponding bulk soil networks in both systems. Network properties and their ecological relevance are described in more detail in Table 1

Bacterial/archaeal networks from conventionally managed soil compartments were more densely connected than the respective networks from organically managed soil compartments, with more edges and higher density despite the same number of nodes (Fig. 5a, Table 1).

**Table 1:** Relevant properties of co-occurrence networks

				Bacterial networks				Fungal networks			
Category	Metric	Definition	Ecological relevance	CB	CR	OB	OR	CB	CR	OB	OR
Size	Nodes	Each node represents a bacterial/archaeal or fungal OTU.	Larger networks contain a greater number of interacting (co-occurring or co-excluding) OTUs.	332	335	335	335	139	142	144	144
Size	Edges	Edges indicate significant co-occurrence or co-exclusion relationships.	Co-occurrence could represent a number of ecological interactions, from predator-prey relationships to commensalism to shared ecological niches [12]. Co-exclusion may represent competition or inhibition.	3698	2995	2088	2261	616	457	669	349
Degree	Mean degree	Degree refers to the number of edges a given node has. Mean degree is the average degree across all nodes in a network [10].	Higher mean degree indicates more co-occurrence or co-exclusion relationships per OTU.	22.28	17.88	12.47	13.50	8.86	6.44	9.29	4.85
Cohesion	Density	Density is defined as the ratio of the number of edges in a given network to the number of edges possible for that many nodes.	High-density networks contain a large proportion of interacting OTUs.	0.067	0.054	0.037	0.040	0.064	0.046	0.065	0.034
Centrality	Centralization index	The degree of organization of a network around specific (central) nodes.	High scores indicate that networks are centralized around one or a few focal nodes; low scores indicate decentralized structure [103].	0.17	0.15	0.11	0.13	0.17	0.10	0.16	0.071
Modularity	Modularity index	Edges belonging to a module minus those that would be expected from a random network with the same number of edges [104].	High modularity indicates more structured communities within a network [104].	0.44	0.49	0.66	0.63	0.45	0.72	0.39	0.77
	Number of modules	Modules are groups of OTUs that interact more closely with one another than with other OTUs.	Can represent overlapping ecological niches or phylogenetic groups [19].	14	9	19	13	18	13	16	13

The bacterial/archaeal network in the conventional bulk soil (CB) was the most densely connected, with nearly 703 more edges than the next-largest network (CR). The bacterial networks were low in density, ranging from 0.037 for OB to 0.067 for CB, and all four networks had significant modularity, with values for the modularity index (range − 0.5 ≤ *Q* ≤ 1) from 0.44 for CB to 0.66 for OB. Significant M × R effects on bacterial communities were reflected in network structure: while the rhizosphere network was smaller, less connected, less dense, and less centralized than the bulk soil network in the conventional system, opposite trends were observed for the organic system.

The impact of management on fungal networks was less clear than for bacteria/archaea (Fig. 5b, Table 1). The fungal network of the organic system bulk soil was largest with 144 nodes and 669 edges and had the highest mean degree and density. Density was low (0.034–0.065), and modularity values ranged from 0.39 for OB to 0.77 for OR. We observed significant rhizosphere effects as fungal rhizosphere networks were smaller, less connected, less dense, less centralized, and more modular than the corresponding bulk soil networks. No M × R interaction was observed in fungal networks.

### Hub taxa

Five hubs were identified in each network as the ASVs with the highest betweenness centrality indices (Additional file 6: Table S5). Normalized betweenness centrality indices were generally lower in the organic networks than the corresponding conventional networks.

As the ecological relevance of hub species in co-occurrence networks has been called into question, particularly with regard to their potential role as keystone species [48], we examined whether any of these taxa also appeared as indicator species. Four bacterial and four fungal hubs were also identified as indicators (Additional file 6: Table S5, bold). Bacterial hubs that were also indicators included members of the orders Sphingobacteriales (CB), Cytophagales (CR), and Rhizobiales (OB), as well as a member of the phylum Verrucomicrobia not identified to the order level (CR). Fungal hubs that were also identified as indicators included members of the orders Tremellales (CB) and Agaricales (CR), as well as a member of the phylum Mortierellomycota that was not identified at the order level and a fungal ASV that could not be identified even at the phylum level.

### Functional N-cycling genes

**Fig. 6:**
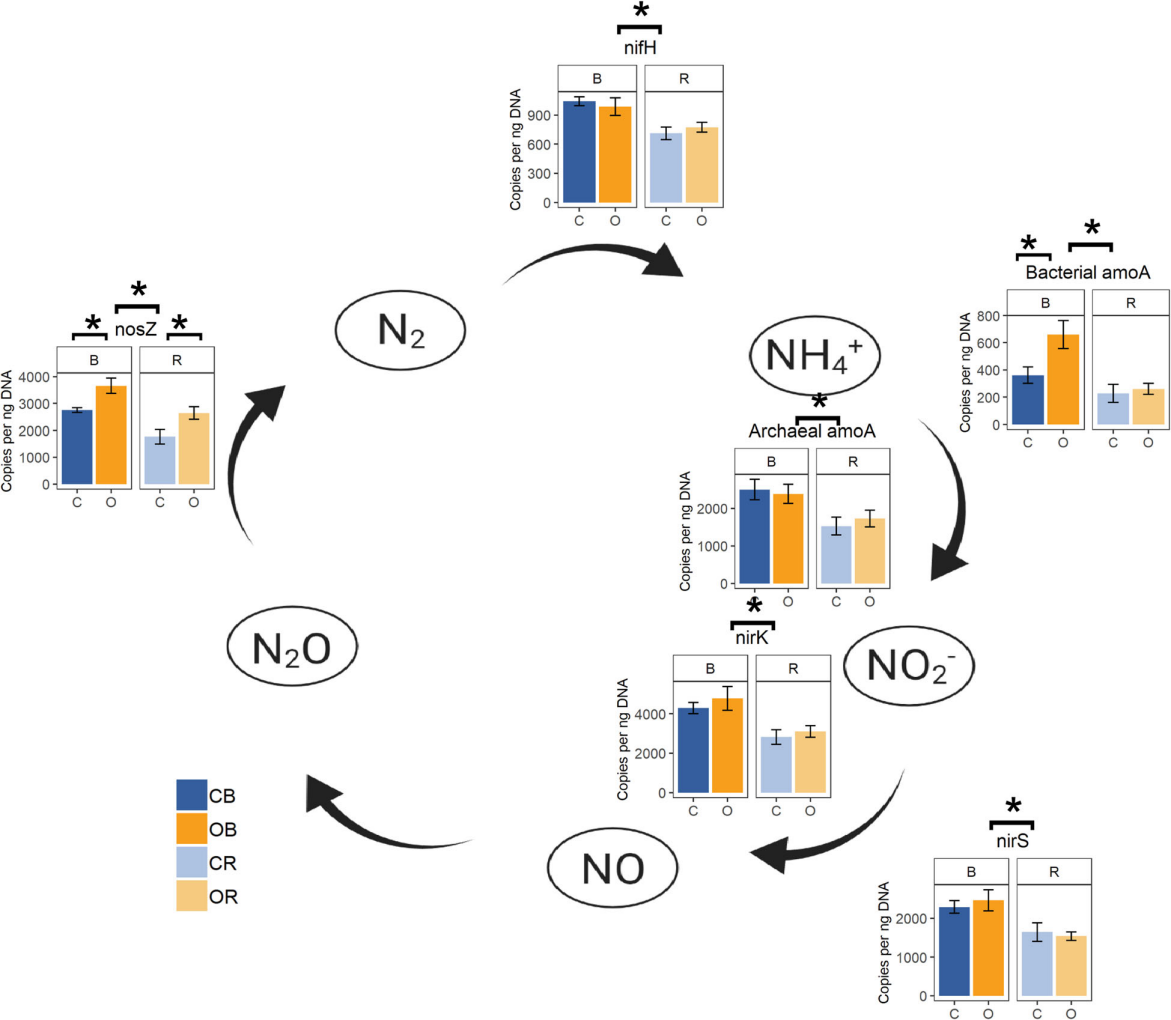
Quantitative PCR of nitrogen-cycling gene abundances. The abundance of six N-cycling genes involved in nitrogen fixation (*nifH*), ammonia oxidation by archaea (archaeal *amoA*) and bacteria (bacterial *amoA*), and denitrification (*nirK*, *nirS*, *nosZ*) across samples. The abundance of all genes was lower in the rhizosphere. Organic management increased the abundance of the *nosZ* gene in both bulk and rhizosphere samples and of the bacterial *amoA* gene in bulk soil. C conventional, O organic, B bulk, R rhizosphere. * indicates a significant difference at the *α* = 0.05 level

Multivariate analysis of all six N-cycling genes showed that samples separated primarily by soil compartment along the first principal component axis, which explained 69.6% of variation (Additional file 11: Figure S4a). Slight separation of samples by management system was also observed along this axis. PERMANOVA revealed significant effects of management (*p* < 0.05) and soil compartment (*p* < 0.001) but not the interaction (*p* > 0.05). This result is consistent with similar profiles of gene abundances across treatments (Additional file 11: Figure S4b). Management effects were detected for abundance of the *nosZ* gene, involved in denitrification, and the bacterial *amoA* gene, involved in nitrification (*p* < 0.05, Fig. 6). The abundance of the *nosZ* gene was higher in the organic system in both bulk and rhizosphere soils, while the abundance of the *amoA* gene was higher in the organic system only in bulk soil. The rhizosphere effect decreased the abundance of all the N-cycling genes measured as compared to bulk soil (Fig. 6). No M × R interactions were significant at the *p* = 0.05 level.

## Discussion

We asked how agricultural management and plant roots act individually and in combination to shape microbial community composition, co-occurrence patterns, and N-cycling functions, and whether this interaction leads to system-specific adaptation. In accordance with known management and rhizosphere effects on microbial community structure and N dynamics in agroecosystems, we observed conventional/organic and bulk/rhizosphere differences in many of the parameters measured. Furthermore, many of our analyses supported the hypothesis that plant selective influence varies with management (an M × R interaction) to shape plant-associated microbial community composition and structure (Fig. 1).

Management, rhizosphere, and M × R effects on microbial communities are likely mediated in large part by soil physicochemical properties, which differed between management systems and soil compartments (Additional file 2: Table S1). Strong effects of management on soil physicochemical properties were visible in the higher NO_3_-N, P, K, Ca, Na, and SOM levels in the organic system and higher Mg and pH in the conventional system. Rhizosphere soil was depleted in NO_3_-N, P, and K in both management systems. M, R, and M × R effects on soil properties such as nutrient availability, pH, and organic matter likely contribute greatly to microbial community assembly in these treatments.

Significant differences in the direction or magnitude of the rhizosphere effect were observed for bacterial diversity, community composition, and indicator species (Additional file 8: Figure S1, Additional file 9: Figure S2, Additional file 10: Figure S3). Plant roots consistently imposed a strong selective filter, and similarity between rhizosphere communities (CR-OR) was greater than similarity between bulk soil communities (CB-OB). Nevertheless, rhizosphere communities still reflected the impacts of management on the contributing microbial pool, and rhizosphere communities were more similar to their corresponding bulk soil communities (CB-CR, OB-OR) than to one another (CR-OR).

The direction of the rhizosphere effect varied with management for bacterial diversity, indicator species, and community structure. This M × R interaction resulted in rhizosphere bacterial communities that were more similar in diversity, composition, and structure than bulk soil bacterial communities. Rhizosphere bacterial/archaeal diversity was lower in the organic rhizosphere but higher in the conventional rhizosphere compared to bulk soil (Additional file 8: Figure S1a). Although roots are often thought to impose a selective filter that decreases diversity, higher species richness in the rhizosphere as observed here in the conventional system has been reported elsewhere when plants select for enrichment of certain processes [49]. Here, however, whether functional enrichment is related to selection for increased diversity is unclear.

Environmental filtering may account for the fact that bacterial rhizosphere networks were more similar than bulk soil networks. Although it has been hypothesized that niche sharing should lead to greater co-occurrence and thus more densely connected networks in the rhizosphere [50], this effect was seen only in the bacterial organic networks (Fig. 5, Table 1). Viewed in combination with previous work showing smaller, less densely connected networks in rhizosphere soil [3, 19–21], our results suggest that rhizosphere effects on co-occurrence networks, like other metrics of microbial community structure, may well be context- and system-dependent.

The magnitude of plant effects on rhizosphere communities also differed between management systems. We generally found greater differences between bulk and rhizosphere community composition in conventional soils compared to organic (Figs. 2, 3, and 4). Hartman et al. attribute a similar M × R interaction observed in their study of wheat agroecosystems to the application of management practices immediately before root establishment [44]. This explanation may apply here as well, specifically with regard to the spatial scale of cover crop and fertilizer inputs. Inorganic fertilizer (conventional system) and composted poultry manure (organic system) were trenched in seed beds and therefore near crop roots, likely favoring divergence of bulk soil and rhizosphere microbial communities. Since cover crops were sown throughout the organic plots, cover-cropping-induced changes in microbial community composition were likely similar in the bulk soil and early root zone, whereas emerging roots in the conventional plots would likely have encountered a fertilizer-enriched zone already distinct from most of the bulk soil.

We further hypothesized that rhizosphere communities would be enriched in system-specific beneficial taxa and functions of importance for plant adaptation to system-specific soil conditions. Although indicator species analysis revealed system-specific taxa, we cannot definitively conclude whether these taxa are beneficial based on amplicon sequencing data. Three members of the order Myxococcales (identified as the genera *Phaselicystis*, *Archangium*, and *Myxococcus*) and two members of the order Burkholderiales (identified as the genera *Rhizobacter* and *Achromobacter*) were indicators of organic environments, in line with previous studies showing these orders to be organic-system-specific [8, 51] (Additional file 4: Table S3). Two strains of the Anaerolineales, an order that displaces other fermenters under high-nitrate conditions [52], were indicators of the conventional system.

Broad ecological information about soil fungi is limited in comparison to bacteria and archaea, despite extensive specialized literature on pathogens of humans and plants or AMF and other endophytes [53]. Many fungal indicators identified here belong to genera known to be pathogenic on other host species, and these were relatively evenly distributed among environments. The significance of pathogens as indicator species in these systems is unclear, especially for pathogens such as *Boeremia exigua*, which causes leaf spot on diverse host crops including tomato, the other crop in this rotation [54], but is not known to cause disease in maize. Fewer details of metabolism and ecology are available for non-pathogenic fungal indicators. *Mortierella*, the most common genus among fungal indicators in this study, are known to be a large genus of saprotrophs [55]. *Exophiala equina* and *Didymella* sp. have been reported elsewhere to be associated with plant roots [56, 57]. Fungi are critical drivers of C/N cycling [58, 59] and carbon sequestration [60] in agricultural systems, and linking specific taxa to roles beyond pathogenic interactions will be a valuable expansion of the existing literature.

With regard to N-cycling functions, we quantified six genes involved in different steps of the nitrogen cycle, all of which were affected by plant selection and only two of which were differentially selected between systems (Fig. 6). The relative abundance of genes relative to one another was similar across treatments, suggesting that no system-specific bottlenecks in the N cycle were observed (Additional file 11: Figure S4b). The abundances of the *nifH*, *amoA* (both archaeal and bacterial), *nirK*, *nirS*, and *nosZ* genes were higher in the bulk soil, in contrast to previous studies that found the maize rhizosphere was enriched in functional genes related to nitrogen fixation (*nifH*), nitrification (*amoA*, *hao*), and denitrification (*narG*, *nirS*/*nirK*, *norB*, *nosZ*) [35–37]. That effect was also observed with the addition of artificial maize root exudates [61], suggesting that exudates are the main mechanisms influencing microbial N cycling independently of other physicochemical characteristics of the rhizosphere. However, mechanisms other than exudates may be responsible for the discrepancy in the direction of the rhizosphere effect between the present study and the literature: while certain root exudates inhibit nitrification in wheat, sorghum, and rice, this effect has not been shown in maize [62]. Sampling in the present study occurred during the silking period of maize, when crop N uptake reaches a maximum. The rhizosphere may be N-depleted in comparison to bulk soil, and microbial N limitation may account for the decreased abundance of these N-cycling genes. Differences in soil organic matter or shifts in root exudation during development [63] leading to altered rhizosphere carbon availability may also account for the change in direction of the rhizosphere effect in the present study as compared to the literature. Increased sampling frequency over the course of the growing season paired with metabolomic analysis of root exudates would provide insight into the mechanisms linking root C release and N uptake dynamics to microbial N-cycling gene abundances.

We hypothesized that differences in N-cycling gene abundance between conventional and organic systems would reflect adaptive shifts, increasing the abundance of gene pathways linking system-specific N inputs to plant-available species, but this hypothesis was not supported. Only two of six genes were affected by soil management history. The abundance of the *nosZ* and bacterial *amoA* genes, the only genes affected by the M × R interaction, was higher in the organic system (Fig. 6). The increase in abundance of the *nosZ* gene could potentially indicate greater conversion of N_2_O to N_2_ and decreased greenhouse gas production [64], while increased abundance of the *amoA* gene may reflect increased conversion of ammonium to nitrite and subsequent nitrification products. Higher soil carbon as a result of long-term organic matter applications at this site [65] may contribute to higher abundances of the *nosZ* gene in bulk and rhizosphere soil in this system. Putz et al. found that higher soil organic carbon under a ley rotation increased expression of the *nrfA* and *nosZ* genes relative to the *nirK* gene as compared to a conventional cereal rotation, favoring higher rates of dissimilatory nitrate reduction to ammonium and lower rates of denitrification [66]. However, previous work in the treatments examined in the present study found that abundances of the *amoA* and *nosZ* genes were not correlated with gross rates of N transformation processes [29]. Prediction of cropping system impacts on microbial N cycling therefore requires a nuanced integration of gene abundances with parameters such as carbon availability, moisture content, and temperature within soil aggregate microenvironments over time. That few differences were observed late in the growing season between N-cycling genes in systems receiving organic or inorganic N inputs is consistent with the results of a meta-analysis by Geisseler and Scow [32], which found that N fertilizer impacts on microbial communities tend to fade over time. Sampling occurred at silking in the present study, long after the preplant fertilizer and compost applications that likely maximize differentiation between systems. Potential N limitation in the rhizosphere in both systems may also have outweighed management effects.

Co-occurrence networks, which provide insight into ecological interactions among microbial taxa [10], were influenced by M, R, and M × R effects. Bulk and rhizosphere bacterial networks from the conventional system had the same number of nodes but were more densely connected than networks from the corresponding soil compartment in the organic system (Fig. 5). Other bulk soil comparisons of organic and conventional agroecosystems using networks constructed from OTU-level data have found conventional networks to have more nodes [13] or, alternatively, fewer nodes and edges than organic networks [14, 15]. Clearly, predicting co-occurrence patterns of incredibly diverse microbial communities based on a conventional-versus-organic classification is too simplistic. Agricultural management is likely better represented as a continuum (or continua along multiple axes) than discrete categories, and causal relationships between specific practices and network topological properties have yet to be determined. An M × R interaction was also observed for network properties in which size, density, and centralization were lower in the rhizosphere network from the conventional system than from the organic system (Fig. 5, Table 1). These network properties follow the same pattern as alpha diversity of bacterial communities, suggesting a shared yet perplexing cause: while the mechanism remains unclear, rhizosphere communities appear to be converging from very distinct bulk soils towards similar diversity and structural metrics. Conventional agriculture is hypothesized to disrupt the connections between bulk soil and rhizosphere networks, as tillage and mineral fertilization are proposed to disturb fungi and soil fauna that serve as a bridge between bulk soil and rhizosphere environments [50]. While tillage does not differ between the systems we measured, fertilization effects are likely partly responsible for the observed interaction. Regardless of the mechanisms involved, the system-specific direction of the rhizosphere effect on co-occurrence network properties suggests that management and plant influence interactively determine not only which taxa are present, but how they interact, with potential implications for agriculturally relevant functions and ecological resilience.

Hub ASVs were identified in each network based on high values for normalized betweenness centrality, a metric often used to describe keystone taxa. Organic networks had lower normalized betweenness centrality values than conventional networks (Additional file 6: Table S5). Lower betweenness centrality values for hub taxa may indicate that network structure depends less on individual species, potentially increasing resilience to environmental stresses that could destabilize networks overly dependent on hub taxa sensitive to those specific stresses. Different hub ASVs were identified in each rhizosphere environment, but information on the ecology of these taxa is generally absent from the literature. Although it would be misleading to state that these taxa are keystone species in their respective habitats without experimental validation [48], the fact that many of these taxa were also identified through indicator species analysis (Additional file 6: Table S5, bold) suggests that they play important ecological roles. Future work could explore the genomes of these ASVs to discern why they are important in their respective agricultural systems and test the hypothesis that they serve as keystone species using synthetic communities.

Concluding whether adaptive plant-microbe feedbacks result in an M × R interaction leading to shifts in other rhizosphere processes is complicated by the importance of poorly understood fungal communities and methodological limitations of this study. Numerous fungal taxa respond to the M × R interaction according to our differential abundance analysis (Fig. 4), yet knowledge of these taxa remains limited due in part to the constraints of culture-dependent methods prevalent in the past. Nonetheless, fungi influence inter-kingdom interactions and agriculturally relevant processes in the rhizosphere [67], and novel molecular biology tools could be used to improve our understanding of key fungal regulators identified in these analyses [68]. Metagenomics and -transcriptomics would facilitate a much more comprehensive analysis of potential functional shifts. A highly useful starting point would be to delve into dynamic variation in microbial genes involved in carbon metabolism and nitrogen cycling in the rhizosphere, in combination with root exudate metabolomics and measurements of root N uptake. Stable isotope labeling and in situ visualization methods [69–73] could further complement our understanding of how management, plant roots, and their interactive effects shape rhizosphere processes.

The scope of this study was intentionally restricted to a single genotype of one crop in two management systems to limit the main sources of variation to the management and rhizosphere effects that were of primary interest, but the limits to inference of this small-scale study must be considered. Other studies in maize have found that strong legacy effects of soil management history are generally acted upon in a similar manner by two maize cultivars [74] and that rhizosphere bacterial community composition varies only slightly among hybrids from different decades of release [75]. Testing whether these limited effects of plant selection hold true for additional contrasting genotypes and genetic groups of maize would further complement this work. Furthermore, variation in root system architecture across crop genotypes might interact with tillage and soil properties responsive to management effects. Management practices such as the inclusion of forage or cover crops planted in stands rather than rows might affect the differentiation of bulk and rhizosphere soil uniquely from systems based on perennial crops, successive plantings of row crops in the same locations, and/or minimal tillage. Study designs incorporating more genotypes, management systems, and cultivation environments would therefore be highly useful to test how results of this study may be extrapolated to other settings.

Future studies should also identify functional genes that are upregulated or downregulated in the rhizosphere under specific agricultural management practices. Whether such functional shifts are adaptive will provide insight into the relationship between agroecology and ecology. Positive eco-evolutionary feedbacks resulting in adaptive microbial communities have been described in unmanaged ecosystems, for example, habitat-adapted symbiosis in saline or arid environments [76, 77]. If similar adaptive recruitment can occur with annual crops in the context of agroecosystems, maximizing this process should be added to the list of rhizosphere engineering strategies and targets for G × E breeding screens [78, 79].

Finally, while our results provide evidence that management and plant influence interact to shape microbial communities at one sampling point, we highlight the need to reframe the M × R interaction as a dynamic process. Rhizosphere communities may be more different from one another than bulk soil communities because roots develop right after tillage and fertilization, when management systems are most distinct [44]. Plants are not static entities, but active participants in the ongoing process of rhizosphere recruitment. As an alternative to the “rhizosphere snapshot,” we propose a “rhizosphere symphony” model that acknowledges the active role of root exudates in orchestrating the composition and function of microbial communities. Altered root exudation during development [63] and in response to water [80] and nutrient limitation [81] can upregulate or downregulate microbial taxa and functions, as a conductor brings together different sections of instruments in turn during a symphony. Although it is unknown whether this plasticity in exudate composition occurs in response to agricultural management, observations of changed exudate quantity and quality in response to soil type [82] (perhaps mediated by the associated microbial communities [83]) and long-term N fertilization [84] suggest that it is possible. Differences in the timing of nutrient availability between management systems, such as delayed N release from cover crop mineralization compared to mineral fertilizer, could thus result in management-system-specific exudate dynamics and rhizosphere microbial communities, i.e., an M × R interaction. If true, this mechanism suggests that we may be able to manipulate the sound of the symphony by talking to the conductor: plant-driven strategies may be instrumental in maximizing beneficial rhizosphere interactions throughout the season.

## Conclusions

Agricultural management and plant selection are known to be powerful influences on microbial community assembly, and our work shows that their interaction results in plant recruitment of management-system-specific taxa and shifts in microbial networks and at least one N-cycling pathway in the rhizosphere. Our finding that agricultural management practices impact rhizosphere microbial communities differently from the bulk soil should be used to guide research priorities and management decisions. The rhizosphere should be recognized as an integral component of sustainable agriculture research that behaves uniquely in comparison to bulk soil. On one hand, plant effects are often neglected in studies investigating how fertilization, tillage, crop rotations, or other management practices affect microbial communities, but valuable insight can be gained from analyzing both bulk and rhizosphere samples. Measuring responses of the bulk soil microbial community can help predict rates of biogeochemical processes at the field, landscape, or ecosystem scale [85, 86]. When plant outcomes such as agricultural productivity are of interest, however, the rhizosphere microbes that are so tightly linked to the health of their host are of critical importance. On the other hand, plant-centric rhizosphere engineering and plant breeding efforts to capitalize on beneficial plant-rhizosphere microbe interactions should not overlook how agricultural management may modify their intended impacts. Understanding and optimizing the interactive effects of management and plant processes regulating rhizosphere assembly provides untapped opportunities to develop more sustainable and productive agroecosystems.

## Methods

### Soil collection and processing

Sampling was conducted during the silking phase of maize (NuTech OA-290 CNV) on July 5, 2017 in the Century Experiment at the Russell Ranch Sustainable Agriculture Facility (Winters, CA, USA). Samples were collected from three plots per treatment (*n* = 6 plots) in the maize-tomato rotations, which have been under continuous organic and conventional management, respectively, for 23 years. Plots were furrow-irrigated and planting density was 80,275 plants ha^− 1^. In each plot, shovels were used to remove three randomly selected maize plants (*n* = 18 plants) and the associated root crowns to a depth of 20 cm. Only plants with adjacent plants on both sides were chosen to avoid edge effects. Samples were taken from two soil compartments (*n* = 36 samples): the rhizosphere and bulk soil. Rhizosphere soil was sampled from the soil adhering to the root crowns, where rooting was so dense that all soil was determined to be under the influence of roots. Bulk soil was taken adjacent to the excavated plant (20 cm from where the stalk had been) from 0 to 20 cm depth. Bulk and rhizosphere samples for DNA analysis were sieved to 2 mm, gravimetric water content was recorded, and samples were stored at − 80 °C. Soil chemical properties were analyzed at the UC Davis Analytical Lab (Davis, CA, USA); soil properties and the corresponding protocol citations can be found in Additional file 2: Table S1.

### Sequencing and bioinformatics

Genomic DNA was extracted from bulk and rhizosphere soil with a DNeasy PowerSoil kit according to manufacturer’s instructions (Qiagen, Inc.) and DNA was stored at − 80 °C. Investigation of microbial communities was based on paired-end amplicon sequencing of the 16S rRNA gene and the ITS region of fungal ribosomal DNA on an Illumina MiSeq PE 300 platform. The 16S rRNA gene was amplified using the primers 515F (5′-GTGCCAGCMGCCGCGGTAA-3′) and 806R (5′-GGACTACHVGGGTWTCTAAT-3′), which are specific to the V4 region [87]. The ITS region was targeted with the primers ITS1F (5′-CTTGGTCATTTAGAGGAAGTAA-3′) and ITS2R (5′-GCTGCGTTCTTCATCGATGC-3′) [88]. Raw data generated from sequencing were demultiplexed using idemp, and primers were removed using cutadapt [89]. All further read processing was done in the dada2 package [90] of R v.3.4.1 [91]. 16S rRNA gene forward reads were truncated to 240 bp and reverse reads to 160 bp based on read quality profiles, and all reads were filtered and trimmed using the parameters maxEE = 2 and truncQ = 2. ITS reads were not truncated to a specific length, as the length of this region is highly variable, and filtering and trimming was done with the parameters maxEE = 2 and truncQ = 11. Bacterial and archaeal taxonomy was assigned to the genus level using the SILVA reference database v.128 [92], and fungal taxonomy was assigned using the 2017 release of the UNITE database [93]. Sequences were rarefied to 4880 reads per sample for bacteria/archaea and 19,438 reads per sample for fungi, leaving a total of 2105 bacterial/archaeal and 754 fungal amplicon sequence variants (ASVs) for further analysis.

### Microbial community analysis

Microbial diversity and community composition were analyzed at the ASV level with the phyloseq [94] and vegan [95] packages in R. The Shannon index was calculated for bacterial and fungal samples at the ASV level as a measure of diversity. The effects of plant selection, management, and their interaction on diversity (Shannon index) were tested using ANOVA with plot as a random effect. Because the interaction between fixed effects was significant, the emmeans package was used to test differences between bulk and rhizosphere samples for each management system [96]. Non-metric multidimensional scaling (NMDS) of Bray-Curtis dissimilarity matrices was used to identify differences between microbial communities sampled from conventional bulk (CB), conventional rhizosphere (CR), organic bulk (OB), and organic rhizosphere (OR) soil. Separate ordinations were carried out for bacterial/archaeal and fungal communities. Effects of management (M), rhizosphere (R), and the M × R interaction on microbial community composition were tested with permutational multivariate analysis of variance (PERMANOVA) based on Bray-Curtis dissimilarity with plot as a random effect. Analysis of similarity (ANOSIM), a rank-based test, was carried out on the pairwise differences CB-OB, CR-OR, CB-CR, and OB-OR to complement PERMANOVA. The Bonferroni adjustment was used to control the family-wise error rate at *α* = 0.05 for the family of four comparisons.

We conducted differential abundance analysis to explore which taxa contributed to this variation in community composition and compare the magnitude of the rhizosphere effect in each system. Differentially abundant taxa occur more frequently in one environment in a pairwise comparison. Differential abundance analysis was carried out using the DESeq2 package in R [97]. Rarefied data were filtered to remove sequences present in fewer than five samples to prevent bias due to low-prevalence taxa. Pairwise comparisons of ASV abundance were carried out between CB-OB (management in bulk soil), CR-OR (management in rhizosphere soil), CB-CR (rhizosphere in conventional system), and OB-OR (rhizosphere in organic system). ASVs were considered to differ significantly in the two environments at a significance level of *p* < 0.0125 based on the Bonferroni correction to control the family-wise error rate at *α* = 0.05.

Indicator species analysis, a distinct and complementary method, was used to identify microbial taxa preferentially associated with a given environment or pair of environments and to determine the direction of the rhizosphere effect in each system. Indicator taxa are defined based on a combination of specificity (occurring in that environment more frequently than other environments) and fidelity (the majority of taxon members are found in that environment) [98]. Differentially abundant and indicator taxa may overlap, as indicator taxa with high specificity for a given environment may be more abundant there, but indicator taxa with high fidelity but low specificity may not show up in differential abundance comparisons. Indicator ASVs significantly associated with one of the four environments (CB, CR, OB, OR) as well as those affected by management (found in CB + CR or OB + OR) and rhizosphere effects (found in CR + OR) were identified using the indicspecies package in R [98]. The IndVal (indicator value) index was calculated for each species-site combination and tested for significance with 999 permutations using the indicspecies::multipatt function [99]. The Bonferroni correction was used to control the family-wise error rate at *α* = 0.05.

### Co-occurrence network analysis

Co-occurrence networks for CB, CR, OB, and OR samples were constructed to provide insight into the structure and putative ecological interactions of microbial communities. In each of these networks, nodes represent ASVs and edges represent significant co-occurrence relationships. Other network properties thought to be ecologically relevant were calculated for each treatment (*n* = 1), including size, mean degree, density, centralization, and modularity (Table 1).

Only sequences present at least ten times and in at least five samples were included in network analyses to prevent loss of specificity and sensitivity [10], for a total of 335 bacterial and 149 fungal ASVs. HabitatCorrectedNetwork, a correction algorithm that accounts for potential habitat filtering effects, was used to construct correlation tables with Python and account for potential effects of combining samples from different plots at the Russell Ranch Sustainable Agriculture Facility [100]. Habitat filtering leads to spurious co-occurrences among taxa that are associated with specific environments, and correlation tables generated without correcting for these effects will result in inflated co-occurrence networks with a high false positive rate [10]. HabitatCorrectedNetwork reduces the false positive rate by correcting each sample for the mean of that subgroup before generating correlations. Co-occurrence networks were constructed from positive Spearman correlations (*ρ* > 0.75 and *p* < 0.05) using a centered log ratio transformation for CB, CR, OB, and OR. Network properties of interest were calculated using the igraph package [101]. Bacterial/archaeal and fungal hub taxa were identified within each network as the five ASVs with the highest betweenness centrality indices [13, 30]. Hub position in a network could indicate a keystone species whose presence is critical to community structure and function, but defining these species as keystones requires experimental validation [102]. Betweenness centrality indices were normalized to allow comparison across networks.

### Quantitative PCR (qPCR)

We were interested in characterizing four major transformations that occur in the nitrogen cycle: nitrogen fixation, nitrification, denitrification, and dissimilatory nitrate reduction to ammonium. We quantified the abundance of genes that represent different components of the nitrogen cycle (*nifH*, *amoA*, *nirK*, *nirS*, and *nosZ*) in DNA extracted from soil samples (Additional file 7: Table S6). For PCR amplification of all functional genes, a microfluidics Fluidigm Gene Expression chip was used to quantify all genes simultaneously. Genes were amplified using the primers described in Additional file 7: Table S6. The thermocycler program was 95 °C for 10 min followed by 14 cycles of 95 °C for 15 s and 58 °C for 4 min. A 5-μL mixture was then prepared with a final concentration of 1X SsoFast EvaGreen Supermix with Low Rox (Bio-Rad Laboratories, Hercules, CA), 1X DNA Binding Dye Sample Loading Reagent (Fluidigm, San Francisco, CA), and 2.25 μl pre-amplified product. A separate master mix was prepared with a final concentration of 1X Assay Loading Reagent (Fluidigm, San Francisco, CA), 0.5X DNA Suspension Buffer (Teknova, Hollister CA), and 50 μM of each forward and reverse primer. Each 5-μL mixture containing product was mixed with 5 μL of master mix and loaded onto a 96.96 Fluidigm Gene Expression chip. Fluidigm amplification was performed according to the following program: 70 °C for 40 min, 58 °C for 30 s, 95 °C for 1 min followed by 30 cycles of 96 °C for 5 s, 58 °C for 20 s, and followed by dissociation curve. Standards for each gene were prepared from sample-derived amplicons from a mixture of soils that were quantified and serially diluted prior to analysis on the Fluidigm system. All samples and standards were analyzed in 12 replicates. Fluidigm Real-Time PCR Analysis software version 4.1.3 and the copy number of each gene (Qubit) were used to determine the C_t_. All Fluidigm RT-qPCR was conducted at the Roy J. Carver Biotechnology Center at the University of Illinois at Urbana-Champaign (Urbana, IL, USA). Fluorescence data were converted to gene copies per ng DNA using standard curves generated individually for each gene from serial dilutions of a corresponding standard of known concentration.

Mean values and standard errors for number of copies per ng DNA were calculated from technical replicates with quality scores of at least 0.65. Technical replicates that were not detected (and thus failed to pass this quality score threshold) were not considered in subsequent analyses. Principal components analysis (PCA) was used to ordinate samples, and PERMANOVA (vegan::adonis) was used to test the fixed effects of management, soil compartment, and their interaction on gene abundance in R [91]. Data were then subset by gene and ANOVA was conducted (mixlm::lmer) on each gene to test fixed effects of management, soil compartment, and their interaction with sampling plot as a random effect. Residuals were tested for normality (stats::shapiro.test), and outliers farther than four times Cook’s distance from the mean were removed until normality of residuals was satisfied (up to two outliers).

## Supplementary information

**Additional file 1.** Raw data from qPCR analysis. This file contains Fluidigm output data for all samples (“Sample ID” column contains abbreviations as used in this manuscript, with biological replicate number following the abbreviation). Twelve technical replicates were prepared for each sample, but only samples that passed the quality control check (see “Call” column) were included in calculations.

**Additional file 2: Table S1.** This file contains Table S1: Soil properties.

**Additional file 3: Table S2.** This file contains Table S2: ANOSIM pairwise comparisons of microbial community composition.

**Additional file 4: Table S3.** This file contains Table S3: Bacterial indicator taxa.

**Additional file 5: Table S4.** This file contains Table S4: Fungal indicator taxa.

**Additional file 6: Table S5.** This file contains Table S5: Hub taxa.

**Additional file 7: Table S6.** This file contains Table S6: Genes quantified using qPCR.

**Additional file 8: Figure S1.** Alpha diversity of bacterial and fungal communities. A) Bacterial diversity was affected by plant selection, but the direction of the effect varied between management systems. B) Fungal diversity was not affected by management or rhizosphere effects. * indicates a significant difference between soil compartments within management system at the α = 0.05 level. Diversity analyses were conducted at the ASV level.

**Additional file 9: Figure S2.** Relative abundances of bacterial and fungal genera. A) Genus-level relative abundance data showed that Bacillus, Skermanella, and Steroidobacter were among the most common bacterial genera in all samples. The genus Pseudarthrobacter tended to be more abundant in bulk than rhizosphere samples in both systems, whereas the genera RB41 and Acidibacter tended to be more abundant in the rhizosphere in both systems. B) The fungal genera Mortierella and Cryptococcus tended to be most abundant across all samples. Members of the genus Cystofilobasidium tended to be more abundant in the organic system, whereas members of the genera Rhizopus and Minimedusa tended to be more abundant in the conventional system. The genera Articulospora and Aspergillus appeared to respond to plant selection, with Articulospora more abundant in bulk soil and Aspergillus more abundant in the rhizosphere. Only the 20 most abundant bacterial and fungal genera are represented with unique colors; all other genera are contained in “Other”.

**Additional file 10: Figure S3.** Indicator species analysis. A) Seventy-four bacterial and B) 49 fungal ASVs were identified as indicator species, occurring often in a given environment and rarely elsewhere. Far more ASVs were management-specific than rhizosphere-specific. Taxonomic information about the indicator ASVs can be found in Additional file 4: Table S3 and Additional file 5: Table S4. C = conventional, O = organic, B = bulk, R = rhizosphere.

**Additional file 11: Figure S4.** Multivariate analysis of N-cycling functional genes. A) Principal components analysis (PCA) revealed that samples separated primarily by soil compartment and secondarily by management system along the first principal component axis, which explained 69.6% of variation. B) Patterns of gene abundances for the N-cycling genes *amoA* (archaeal and bacterial), *nifH*, *nirK*, *nirS*, and *nosZ* were similar for all treatments, suggesting that system-specific bottlenecks in the N cycle were not observed. Accordingly, PERMANOVA revealed significant effects of soil compartment (*p* < 0.001) and management (*p* < 0.05) but not their interaction (*p* > 0.05).

## Data Availability

The dataset supporting the conclusions of this article is available in the NCBI Sequence Read Archive repository, as part of the BioProject Accession PRJNA534086 (https://www.ncbi.nlm.nih.gov/sra/PRJNA534086). The qPCR dataset is available as additional material for this manuscript (see Additional file 1).

## Abbreviations

ASV: Amplicon sequence variant
M: Management effects
N: Nitrogen
R: Rhizosphere effects

## References

[1] Francioli D, Schulz E, Lentendu G, Wubet T, Buscot F, Reitz T (2016). Mineral vs. organic amendments: microbial community structure, activity and abundance of agriculturally relevant microbes are driven by long-term fertilization strategies. Terr Microbiol.

[2] Hinsinger P, Gobran GR, Gregory PJ, Wenzel WW (2005). Rhizosphere geometry and heterogeneity arising from root-mediated physical and chemical processes. New Phytol.

[3] Fan K, Cardona C, Li Y, Shi Y, Xiang X, Shen C (2017). Rhizosphere-associated bacterial network structure and spatial distribution differ significantly from bulk soil in wheat crop fields. Soil Biol Biochem.

[4] Hayat R, Ali S, Amara U, Khalid R, Ahmed I (2010). Soil beneficial bacteria and their role in plant growth promotion: a review. Ann Microbiol.

[5] Lupatini M, Korthals GW, de Hollander M, Janssens TKS, Kuramae EE (2017). Soil microbiome is more heterogeneous in organic than in conventional farming system. Front Microbiol.

[6] Mader P, Fliessbach A, Dubois D, Gunst L, Fried P, Niggli U (2002). Soil fertility and biodiversity in organic farming. Sci Wash C.

[7] Li F, Chen L, Zhang J, Yin J, Huang S (2017). Bacterial community structure after long-term organic and inorganic fertilization reveals important associations between soil nutrients and specific taxa involved in nutrient transformations. Front Microbiol.

[8] Wang W, Wang H, Feng Y, Wang L, Xiao X, Xi Y (2016). Consistent responses of the microbial community structure to organic farming along the middle and lower reaches of the Yangtze River. Sci Rep.

[9] Coyte KZ, Schluter J, Foster KR (2015). The ecology of the microbiome: networks, competition, and stability. Science.

[10] Berry D, Widder S (2014). Deciphering microbial interactions and detecting keystone species with co-occurrence networks. Front Microbiol.

[11] Layeghifard M, Hwang DM, Guttman DS (2017). Disentangling interactions in the microbiome: a network perspective. Trends Microbiol.

[12] Faust K, Raes J (2012). Microbial interactions: from networks to models. Nat Rev Microbiol.

[13] Banerjee S, Kirkby CA, Schmutter D, Bissett A, Kirkegaard JA, Richardson AE (2016). Network analysis reveals functional redundancy and keystone taxa amongst bacterial and fungal communities during organic matter decomposition in an arable soil. Soil Biol Biochem.

[14] Xue C, Penton CR, Zhu C, Chen H, Duan Y, Peng C (2018). Alterations in soil fungal community composition and network assemblage structure by different long-term fertilization regimes are correlated to the soil ionome. Biol Fertil Soils.

[15] Ling N, Zhu C, Xue C, Chen H, Duan Y, Peng C (2016). Insight into how organic amendments can shape the soil microbiome in long-term field experiments as revealed by network analysis. Soil Biol Biochem.

[16] Turner TR, Ramakrishnan K, Walshaw J, Heavens D, Alston M, Swarbreck D (2013). Comparative metatranscriptomics reveals kingdom level changes in the rhizosphere microbiome of plants. ISME J.

[17] Peiffer JA, Spor A, Koren O, Jin Z, Tringe SG, Dangl JL (2013). Diversity and heritability of the maize rhizosphere microbiome under field conditions. Proc Natl Acad Sci U S A.

[18] Hamonts K, Trivedi P, Garg A, Janitz C, Grinyer J, Holford P (2018). Field study reveals core plant microbiota and relative importance of their drivers. Environ Microbiol.

[19] Zhang B, Zhang J, Liu Y, Shi P, Wei G (2018). Co-occurrence patterns of soybean rhizosphere microbiome at a continental scale. Soil Biol Biochem.

[20] Fan K, Weisenhorn P, Gilbert JA, Chu H (2018). Wheat rhizosphere harbors a less complex and more stable microbial co-occurrence pattern than bulk soil. Soil Biol Biochem.

[21] Mendes LW, Kuramae EE, Navarrete AA, van Veen JA, Tsai SM (2014). Taxonomical and functional microbial community selection in soybean rhizosphere. ISME J.

[22] Shi S, Nuccio EE, Shi ZJ, He Z, Zhou J, Firestone MK (2016). The interconnected rhizosphere: high network complexity dominates rhizosphere assemblages. Ecol Lett.

[23] Meyer A, Focks A, Radl V, Keil D, Welzl G, Schoening I (2013). Different land use intensities in grassland ecosystems drive ecology of microbial communities involved in nitrogen turnover in soil. Plos One.

[24] Thompson KA, Bent E, Abalos D, Wagner-Riddle C, Dunfleld KE (2016). Soil microbial communities as potential regulators of in situ N2O fluxes in annual and perennial cropping systems. Soil Biol Biochem.

[25] Vermue A, Philippot L, Munier-Jolain N, Henault C, Nicolardot B (2013). Influence of integrated weed management system on N-cycling microbial communities and N2O emissions. Plant Soil.

[26] Wakelin SA, Gregg AL, Simpson RJ, Li GD, Riley IT, McKay AC (2009). Pasture management clearly affects soil microbial community structure and N-cycling bacteria. Pedobiologia.

[27] Bhowmik A, Cloutier M, Ball E, Bruns MA (2017). Underexplored microbial metabolisms for enhanced nutrient recycling in agricultural soils. Aims Microbiol.

[28] Tatti E, Goyer C, Zebarth BJ, Burton DL, Giovannetti L, Viti C (2013). Short-term effects of mineral and organic fertilizer on denitrifiers, nitrous oxide emissions and denitrification in long-term amended vineyard soils. Soil Sci Soc Am J.

[29] Kong AYY, Hristova K, Scow KM, Six J (2010). Impacts of different N management regimes on nitrifier and denitrifier communities and N cycling in soil microenvironments. Soil Biol Biochem.

[30] Gu Y, Wang Y, Lu S, Xiang Q, Yu X, Zhao K (2017). Long-term fertilization structures bacterial and archaeal communities along soil depth gradient in a paddy soil. Front Microbiol.

[31] Muema EK, Cadisch G, Musyoki MK, Rasche F (2016). Dynamics of bacterial and archaeal amoA gene abundance after additions of organic inputs combined with mineral nitrogen to an agricultural soil. Nutr Cycl Agroecosystems.

[32] Geisseler D, Scow KM (2014). Long-term effects of mineral fertilizers on soil microorganisms – a review. Soil Biol Biochem.

[33] Kaštovská E, Edwards K, Picek T, Šantrůčková H (2015). A larger investment into exudation by competitive versus conservative plants is connected to more coupled plant–microbe N cycling. Biogeochemistry.

[34] Cheneby D, Perrez S, Devroe C, Hallet S, Couton Y, Bizouard F (2004). Denitrifying bacteria in bulk and maize-rhizospheric soil: diversity and N2O-reducing abilities. Can J Microbiol.

[35] Li X, Rui J, Xiong J, Li J, He Z, Zhou J (2014). Functional potential of soil microbial communities in the maize rhizosphere. PLoS One San Franc.

[36] Ai C, Liang G, Sun J, Wang X, He P, Zhou W (2013). Different roles of rhizosphere effect and long-term fertilization in the activity and community structure of ammonia oxidizers in a calcareous fluvo-aquic soil. Soil Biol Biochem.

[37] Wang C, Zheng M, Song W, Wen S, Wang B, Zhu C (2017). Impact of 25 years of inorganic fertilization on diazotrophic abundance and community structure in an acidic soil in southern China. Soil Biol Biochem.

[38] Bowles TM, Hollander AD, Steenwerth K, Jackson LE (2015). Tightly-coupled plant-soil nitrogen cycling: comparison of organic farms across an agricultural landscape. PLoS One.

[39] Ollivier J, Töwe S, Bannert A, Hai B, Kastl E-M, Meyer A (2011). Nitrogen turnover in soil and global change. FEMS Microbiol Ecol.

[40] Wilbois K-P, Schmidt JE (2019). Reframing the debate surrounding the yield gap between organic and conventional farming. Agronomy.

[41] Granzow S, Kaiser K, Wemheuer B, Pfeiffer B, Daniel R, Vidal S, et al. The effects of cropping regimes on fungal and bacterial communities of wheat and faba bean in a greenhouse pot experiment differ between plant species and compartment. front Microbiol. 2017;8 Available from: https://www.ncbi.nlm.nih.gov/pmc/articles/PMC5447230/. 10.3389/fmicb.2017.00902 PMC5447230 28611735

[42] Marongiu R, Garau G, Caredda M, Deiana P (2006). Impact of soil management on the functional activity of microbial communities associated to cork oak rhizosphere.

[43] Banerjee S, Walder F, Büchi L, Meyer M, Held AY, Gattinger A, et al. Agricultural intensification reduces microbial network complexity and the abundance of keystone taxa in roots. ISME J. 2019;13:1722–36. 10.1038/s41396-019-0383-2 PMC6591126 30850707

[44] Hartman K, van der Heijden MGA, Wittwer RA, Banerjee S, Walser J-C, Schlaeppi K. Cropping practices manipulate abundance patterns of root and soil microbiome members paving the way to smart farming. Microbiome. 2018. Cited 2018 Feb 1; 6. Available from: https://www.ncbi.nlm.nih.gov/pmc/articles/PMC5771023/10.1186/s40168-017-0389-9 PMC5771023 29338764

[45] Hartwig UA (1998). The regulation of symbiotic N2 fixation: a conceptual model of N feedback from the ecosystem to the gene expression level. Perspect Plant Ecol Evol Syst.

[46] Zhalnina K, Louie KB, Hao Z, Mansoori N, da Rocha UN, Shi S (2018). Dynamic root exudate chemistry and microbial substrate preferences drive patterns in rhizosphere microbial community assembly. Nat Microbiol.

[47] Revillini D, Gehring CA, Johnson NC (2016). The role of locally adapted mycorrhizas and rhizobacteria in plant–soil feedback systems. Funct Ecol.

[48] Röttjers L, Faust K (2019). Can we predict keystones?. Nat Rev Microbiol.

[49] Yan Y, Kuramae EE, de Hollander M, Klinkhamer PGL, van Veen JA (2017). Functional traits dominate the diversity-related selection of bacterial communities in the rhizosphere. Isme J.

[50] de Vries FT, Wallenstein MD (2017). Below-ground connections underlying above-ground food production: a framework for optimising ecological connections in the rhizosphere. J Ecol.

[51] Yin C, Fan F-L, Li Z-J, Song A-L, Zhu P, Peng C (2012). Influences of long-term application of organic and inorganic fertilizers on the composition and abundance of nirS-type denitrifiers in black soil. Huan Jing Ke Xue Huanjing Kexue.

[52] Grießmeier V, Bremges A, McHardy AC, Gescher J (2017). Investigation of different nitrogen reduction routes and their key microbial players in wood chip-driven denitrification beds. Sci Rep.

[53] Ellouze W, Esmaeili Taheri A, Bainard LD, Yang C, Bazghaleh N, Navarro-Borrell A (2014). Soil fungal resources in annual cropping systems and their potential for management. Biomed Res Int.

[54] Hemmati R (2014). First report of Boeremia exigua on tomato in Iran. Iran J Plant Pathol.

[55] Yadav DR, Kim SW, Adhikari M, Um YH, Kim HS, Kim C (2015). Three new records of Mortierella species isolated from crop field soil in Korea. Mycobiology.

[56] Maciá-Vicente JG, Glynou K, Piepenbring M (2016). A new species of Exophiala associated with roots. Mycol Prog.

[57] Chen Q, Hou LW, Duan WJ, Crous PW, Cai L (2017). Didymellaceae revisited. Stud Mycol.

[58] Waring BG, Averill C, Hawkes CV (2013). Differences in fungal and bacterial physiology alter soil carbon and nitrogen cycling: insights from meta-analysis and theoretical models. Ecol Lett.

[59] Malik AA, Chowdhury S, Schlager V, Oliver A, Puissant J, Vazquez PGM (2016). Soil fungal:bacterial ratios are linked to altered carbon cycling. Front Microbiol.

[60] Six J, Frey SD, Thiet RK, Batten KM (2006). Bacterial and fungal contributions to carbon sequestration in agroecosystems. Soil Sci Soc Am J.

[61] Henry S, Texier S, Hallet S, Bru D, Dambreville C, Cheneby D (2008). Disentangling the rhizosphere effect on nitrate reducers and denitrifiers: insight into the role of root exudates. Environ Microbiol.

[62] Coskun D, Britto DT, Shi W, Kronzucker HJ (2017). How plant root exudates shape the nitrogen cycle. Trends Plant Sci.

[63] Boeuf-Tremblay V, Plantureux S, Guckert A (1995). Influence of mechanical impedance on root exudation of maize seedlings at two development stages. Plant Soil.

[64] Krause H-M, Thonar C, Eschenbach W, Well R, Mäder P, Behrens S (2017). Long term farming systems affect soils potential for N2O production and reduction processes under denitrifying conditions. Soil Biol Biochem.

[65] Wolf KM, Torbert EE, Bryant D, Burger M, Denison RF, Herrera I (2018). The century experiment: the first twenty years of UC Davis’ Mediterranean agroecological experiment. Ecology.

[66] Putz M, Schleusner P, Rutting T, Hallin S (2018). Relative abundance of denitrifying and DNRA bacteria and their activity determine nitrogen retention or loss in agricultural soil. Soil Biol Biochem.

[67] Larsen J, Jaramillo-López P, Nájera-Rincon M, González-Esquivel CE (2015). Biotic interactions in the rhizosphere in relation to plant and soil nutrient dynamics. J Soil Sci Plant Nutr.

[68] Singh BK, Millard P, Whiteley AS, Murrell JC (2004). Unravelling rhizosphere–microbial interactions: opportunities and limitations. Trends Microbiol.

[69] Gougoulias C, Meade A, Shaw LJ (2018). Apportioning bacterial carbon source utilization in soil using C-14 isotope analysis of FISH-targeted bacterial populations sorted by fluorescence activated cell sorting (FACS): C-14-FISH-FACS. Environ Microbiol Rep.

[70] Oburger E, Schmidt H (2016). New methods to unravel rhizosphere processes. Trends Plant Sci.

[71] Alony A, Linker R (2013). Development of a laser-induced fluorescence imaging system for root activity and rhizosphere visualisation. Biosyst Eng.

[72] Rogers SW, Moorman TB, Ong SK (2007). Fluorescent in situ hybridization and micro-autoradiography applied to ecophysiology in soil. Soil Sci Soc Am J.

[73] Wallenstein MD, Weintraub MN (2008). Emerging tools for measuring and modeling the in situ activity of soil extracellular enzymes. Soil Biol Biochem.

[74] Bakker MG, Chaparro JM, Manter DK, Vivanco JM (2015). Impacts of bulk soil microbial community structure on rhizosphere microbiomes of Zea mays. Plant Soil.

[75] Emmett Bryan D., Buckley Daniel H., Smith Margaret E., Drinkwater Laurie E. (2018). Eighty years of maize breeding alters plant nitrogen acquisition but not rhizosphere bacterial community composition. Plant and Soil.

[76] Rodriguez RJ, Henson J, Van Volkenburgh E, Hoy M, Wright L, Beckwith F (2008). Stress tolerance in plants via habitat-adapted symbiosis. Isme J.

[77] Rodriguez RJ, Woodward C, Kim Y-O, Redman RS, White JF, Torres MS (2009). Habitat-adapted symbiosis as a defense against abiotic and biotic stresses. Defensive mutual microb symbiosis.

[78] Ryan PR, Dessaux Y, Thomashow LS, Weller DM (2009). Rhizosphere engineering and management for sustainable agriculture. Plant Soil.

[79] Dessaux Y, Grandclément C, Faure D (2016). Engineering the rhizosphere. Trends Plant Sci.

[80] Naylor D, Coleman-Derr D (2018). Drought stress and root-associated bacterial communities. Front Plant Sci.

[81] Dakora FD, Phillips DA (2002). Root exudates as mediators of mineral acquisition in low-nutrient environments. Plant Soil.

[82] Neumann G, Bott S, Ohler MA, Mock H-P, Lippmann R, Grosch R (2014). Root exudation and root development of lettuce (*Lactuca sativa* L. cv. Tizian) as affected by different soils. Front Microbiol.

[83] Schreiter S, Ding G-C, Heuer H, Neumann G, Sandmann M, Grosch R (2014). Effect of the soil type on the microbiome in the rhizosphere of field-grown lettuce. Front Microbiol.

[84] Wang Q, Ma M, Jiang X, Zhou B, Guan D, Cao F (2019). Long-term N fertilization altered 13C-labeled fungal community composition but not diversity in wheat rhizosphere of Chinese black soil. Soil Biol Biochem.

[85] McGuire KL, Treseder KK (2010). Microbial communities and their relevance for ecosystem models: decomposition as a case study. Soil Biol Biochem.

[86] Graham EB, Knelman JE, Schindlbacher A, Siciliano S, Breulmann M, Yannarell A, et al. Microbes as engines of ecosystem function: when does community structure enhance predictions of ecosystem processes? Front Microbiol. 2016. Cited 2019 Sep 17; 7. Available from: https://www.ncbi.nlm.nih.gov/pmc/articles/PMC4764795/10.3389/fmicb.2016.00214 PMC4764795 26941732

[87] 16S Illumina Amplicon Protocol: Earth Microbiome Project. Cited 2018 Jan 12. Available from: http://press.igsb.anl.gov/earthmicrobiome/protocols-and-standards/16s/

[88] Earth Microbiome Project. ITS Illumina Amplicon Protocol: Earth Microbiome Project. Cited 2018 Feb 15. Available from: http://www.earthmicrobiome.org/protocols-and-standards/its/

[89] Martin M (2011). Cutadapt removes adapter sequences from high-throughput sequencing reads. EMBnet J.

[90] Callahan BJ, McMurdie PJ, Rosen MJ, Han AW, Johnson AJA, Holmes SP (2016). DADA2: high-resolution sample inference from Illumina amplicon data. Nat Methods.

[91] R Core Team (2018). R: a language and environment for statistical computing.

[92] Glöckner FO, Yilmaz P, Quast C, Gerken J, Beccati A, Ciuprina A (2017). 25 years of serving the community with ribosomal RNA gene reference databases and tools. J Biotechnol.

[93] Kõljalg U, Nilsson RH, Abarenkov K, Tedersoo L, Taylor AFS, Bahram M (2013). Towards a unified paradigm for sequence-based identification of fungi. Mol Ecol.

[94] McMurdie PJ, Holmes S (2013). phyloseq: an R package for reproducible interactive analysis and graphics of microbiome census data. PLoS One.

[95] Oksanen J, Blanchet FG, Friendly M, Kindt R, Legendre P, McGlinn D, et al. Vegan: community ecology package. 2018. Cited 2018 Feb 9. Available from: https://cran.r-project.org/web/packages/vegan/index.html

[96] Lenth R (2019). emmeans: estimated marginal means, aka least-squares means.

[97] Love MI, Huber W, Anders S (2014). Moderated estimation of fold change and dispersion for RNA-seq data with DESeq2. Genome Biol.

[98] Dufrene M, Legendre P (1997). Species assemblages and indicator species: the need for a flexible asymmetrical approach. Ecol Monogr Durh.

[99] De Cáceres M, Legendre P (2009). Associations between species and groups of sites: indices and statistical inference. Ecology.

[100] Brisson VL, Schmidt JE, Northen TR, Vogel JP, Gaudin A. A new method to correct for habitat filtering in microbial correlation networks. Frontiers in Microbiology. 2018;10:585. 10.3389/fmicb.2019.00585 10.3389/fmicb.2019.00585 PMC6435493 30949160

[101] Csárdi G, Nepusz T. The igraph software package for complex network research. Inter J Complex Syst. 2006. Cited 2018 Feb 9; 1695. Available from: http://igraph.sf.net

[102] Röttjers L, Faust K (2018). From hairballs to hypotheses–biological insights from microbial networks. FEMS Microbiol Rev.

[103] Wasserman S, Faust K. Social network analysis: methods and applications. Cambridge: Cambridge University Press; 1994.

[104] Newman MEJ (2006). Modularity and community structure in networks. Proc Natl Acad Sci.

