# Supplementary material for: Agricultural management and plant selection interactively affect rhizosphere microbial community structure and nitrogen cycling
Source: Microbiome. 2019 Nov 7;7:146. doi: 10.1186/s40168-019-0756-9 (PMC6839119; doi:10.1186/s40168-019-0756-9)
Supplement: Supplementary file 2 — Additional file 2: Table S1. This file contains Table S1: Soil properties. [file 40168_2019_756_MOESM2_ESM.docx]

| Treatment | NO_3_-N | Olsen-P | X-K | X-K | X-Na | X-Na | X-Ca | X-Mg | CEC (estimated) | OM (LOI) | pH |
| --- | --- | --- | --- | --- | --- | --- | --- | --- | --- | --- | --- |
|  | *ppm* | *ppm* | *ppm* | *meq/100g* | *ppm* | *meq/100g* | *meq/100g* | *meq/100g* | *meq/100g* | *%* |  |
| *Protocol reference* | [1,2] | [3,4] | [5] | [5] | [5] | [5] | [5] | [5] | [5] | [6] | [7] |
| Conventional Bulk | 33.3 | 22.7 | 206.5 | 0.53 | 25.5 | 0.11 | 9.13 | 13.6 | 23.3 | 2.34 | 7.15 |
| Conventional Rhizosphere | 8.5 | 20.6 | 141.0 | 0.36 | 35.0 | 0.15 | 9.09 | 13.5 | 23.1 | 2.43 | 7.13 |
| Organic Bulk | 64.5 | 44.4 | 393.0 | 1.01 | 57.0 | 0.25 | 11.35 | 13.1 | 25.7 | 3.43 | 6.98 |
| Organic Rhizosphere | 7.3 | 35.0 | 288.0 | 0.74 | 62.5 | 0.27 | 10.88 | 12.7 | 24.6 | 3.32 | 6.96 |

Table S1: Soil properties

References:

1. Hofer S. Determination of Ammonia (Salicylate) in 2M KCl soil extracts by Flow Injection Analysis. Loveland, CO: Lachat Instruments; 2003. Report No.: QuikChem Method 12-107-06-2-A.

2. Knepel K. Determination of Nitrate in 2M KCl soil extracts by Flow Injection Analysis. QuikChem Method 12-107-04-1-B. Loveland, CO: Lachat Instruments; 2003.

3. Prokopy W. Phosphorus in 0.5 M sodium bicarbonate soil extracts. Milwaukee, WI: Lachat Instruments; 1995. Report No.: QuikChem Method 12-115-01-1-B.

4. Olsen SR, Sommers LE. Phosphorus. In: Page AL, editor. Methods of soil analysis: Part 2 Chemical and microbiological properties. Madison, WI: ASA and SSSA; 1982. p. 1035–49.

5. Thomas GW. Exchangeable cations. In: Page AL, editor. Methods of soil analysis: Part 2 Chemical and microbiological properties. ASA; 1982. p. 159–65.

6. Nelson DW, Sommers LE. Total Carbon, Organic Carbon, and Organic Matter. In: Bigham JM, editor. Methods of Soil Analysis Part 3 Chemical Methods. Madison, WI: SSSA; 1996. p. 1001–6.

7. U.S. Salinity Laboratory Staff. pH reading of saturated soil paste. In: Richards LA, editor. Diagnosis and improvement of saline and alkali soils. Washington, D.C.: U.S. Government Printing Office; 1954. p. 102.
