# Supplementary material for: Agricultural management and plant selection interactively affect rhizosphere microbial community structure and nitrogen cycling
Source: Microbiome. 2019 Nov 7;7:146. doi: 10.1186/s40168-019-0756-9 (PMC6839119; doi:10.1186/s40168-019-0756-9)
Supplement: Supplementary file 3 — Additional file 3: Table S2. This file contains Table S2: ANOSIM pairwise comparisons of microbial community composition. [file 40168_2019_756_MOESM3_ESM.docx]

|  | Comparison | Effect | R statistic | Significance |
| --- | --- | --- | --- | --- |
| Bacteria (16S) | CB-OB | M | 0.77 | 0.001 |
|  | CB-CR | R | 0.54 | 0.001 |
|  | OB-OR | M | 0.28 | 0.007 |
|  | CR-OR | M x R | 0.73 | 0.001 |
| Fungi  (ITS) | CB-OB | M | 0.53 | 0.001 |
|  | CB-CR | R | 0.01 | 0.35 |
|  | OB-OR | M | 0.11 | 0.04 |
|  | CR-OR | M x R | 0.49 | 0.001 |

Table S2: ANOSIM pairwise comparisons of microbial community composition

Abbreviations: C=Conventional, O= Organic R=Rhizosphere, B= Bulk. R statistics represent difference of mean ranks between the two groups. Values closer to 1.0 indicate greater dissimilarity between the two groups compared.
