# Supplementary material for: Agricultural management and plant selection interactively affect rhizosphere microbial community structure and nitrogen cycling
Source: Microbiome. 2019 Nov 7;7:146. doi: 10.1186/s40168-019-0756-9 (PMC6839119; doi:10.1186/s40168-019-0756-9)
Supplement: Supplementary file 7 — Additional file 7: Table S6. This file contains Table S6: Genes quantified using qPCR. [file 40168_2019_756_MOESM7_ESM.docx]

Table S6: Genes quantified using qPCR

| Gene | N-cycling step | F primer | R primer | Reference |
| --- | --- | --- | --- | --- |
| *nifH* | N fixation | PolF | PolR | [1] |
| *amoA* | Archaeal nitrification | CrenamoA23f | CrenamoA6161r | [2] |
| *amoA* | Bacterial nitrification | amoA-1F | amoA-2R | [3] |
| *nirK* | Denitrification | nirK876 | nirK1040 | [4] |
| *nirS* | Denitrification | nirSCd3aF | nirSR3cd | [5] |
| *nosZ* | Denitrification | nosZ1F | nosZ1R | [6] |

1. Poly F, Ranjard L, Nazaret S, Gourbière F, Monrozier LJ. Comparison of nifH Gene Pools in Soils and Soil Microenvironments with Contrasting Properties. Appl Environ Microbiol. 2001;67:2255–62.

2. Tourna M, Freitag TE, Nicol GW, Prosser JI. Growth, activity and temperature responses of ammonia-oxidizing archaea and bacteria in soil microcosms. Environ Microbiol. 2008;10:1357–64.

3. Rotthauwe JH, Witzel KP, Liesack W. The ammonia monooxygenase structural gene amoA as a functional marker: molecular fine-scale analysis of natural ammonia-oxidizing populations. Appl Environ Microbiol. 1997;63:4704–12.

4. Henry S, Baudoin E, López-Gutiérrez JC, Martin-Laurent F, Brauman A, Philippot L. Quantification of denitrifying bacteria in soils by nirK gene targeted real-time PCR. J Microbiol Methods. 2004;59:327–35.

5. Kandeler E, Deiglmayr K, Tscherko D, Bru D, Philippot L. Abundance of narG, nirS, nirK, and nosZ Genes of Denitrifying Bacteria during Primary Successions of a Glacier Foreland. Appl Environ Microbiol. 2006;72:5957–62.

6. Henry S, Bru D, Stres B, Hallet S, Philippot L. Quantitative Detection of the nosZ Gene, Encoding Nitrous Oxide Reductase, and Comparison of the Abundances of 16S rRNA, narG, nirK, and nosZ Genes in Soils. Appl Environ Microbiol. 2006;72:5181–9.
