# Supplementary figures and images for: Agricultural management and plant selection interactively affect rhizosphere microbial community structure and nitrogen cycling
Source: Microbiome. 2019 Nov 7;7:146. doi: 10.1186/s40168-019-0756-9 (PMC6839119; doi:10.1186/s40168-019-0756-9)

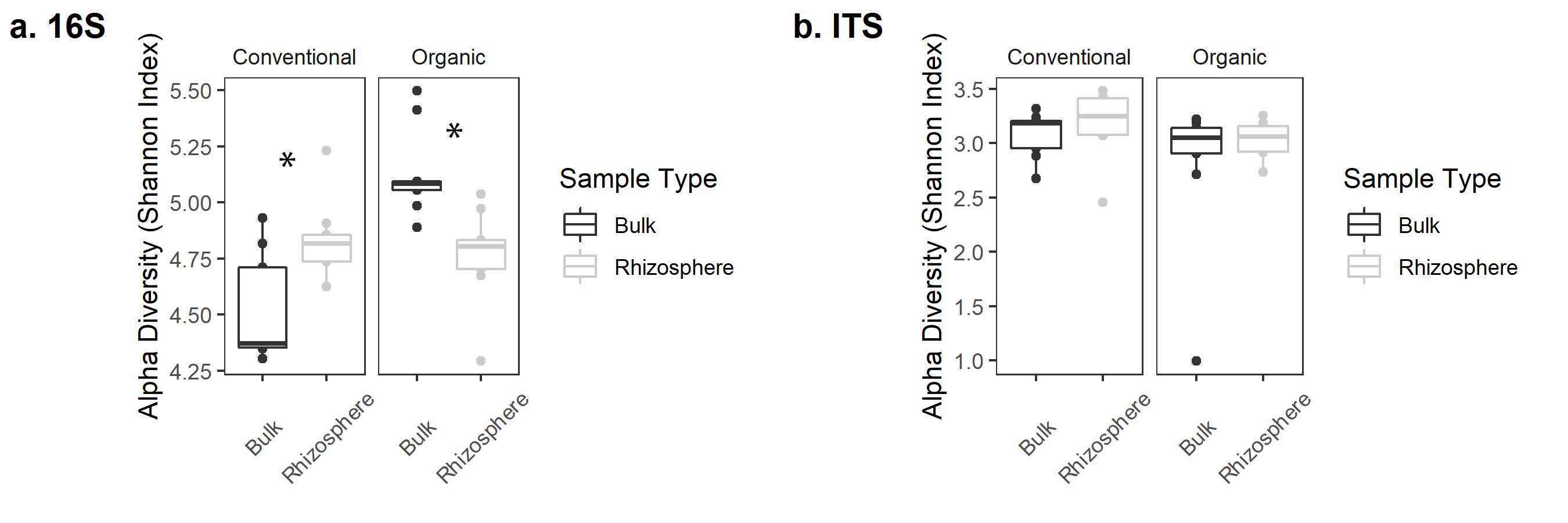

Supplement: Supplementary file 8 — Additional file 8: Figure S1. Alpha diversity of bacterial and fungal communities. A) Bacterial diversity was affected by plant selection, but the direction of the effect varied between management systems. B) Fungal diversity was not affected by management or rhizosphere effects. * indicates a significant difference between soil compartments within management system at the α = 0.05 level. Diversity analyses were conducted at the ASV level. [file 40168_2019_756_MOESM8_ESM.tif]

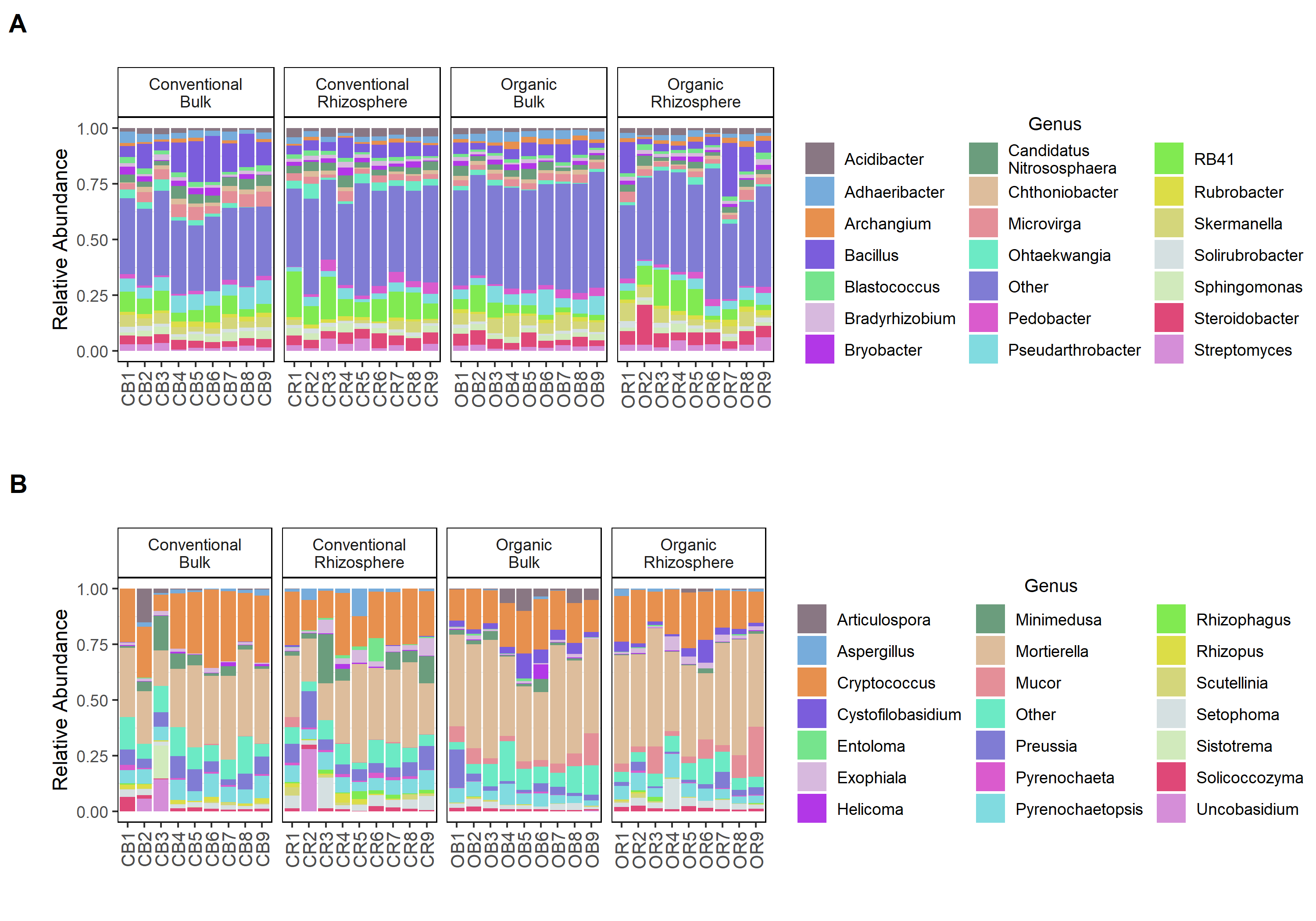

Supplement: Supplementary file 9 — Additional file 9: Figure S2. Relative abundances of bacterial and fungal genera. A) Genus-level relative abundance data showed that Bacillus, Skermanella, and Steroidobacter were among the most common bacterial genera in all samples. The genus Pseudarthrobacter tended to be more abundant in bulk than rhizosphere samples in both systems, whereas the genera RB41 and Acidibacter tended to be more abundant in the rhizosphere in both systems. B) The fungal genera Mortierella and Cryptococcus tended to be most abundant across all samples. Members of the genus Cystofilobasidium tended to be more abundant in the organic system, whereas members of the genera Rhizopus and Minimedusa tended to be more abundant in the conventional system. The genera Articulospora and Aspergillus appeared to respond to plant selection, with Articulospora more abundant in bulk soil and Aspergillus more abundant in the rhizosphere. Only the 20 most abundant bacterial and fungal genera are represented with unique colors; all other genera are contained in “Other”. [file 40168_2019_756_MOESM9_ESM.tif]

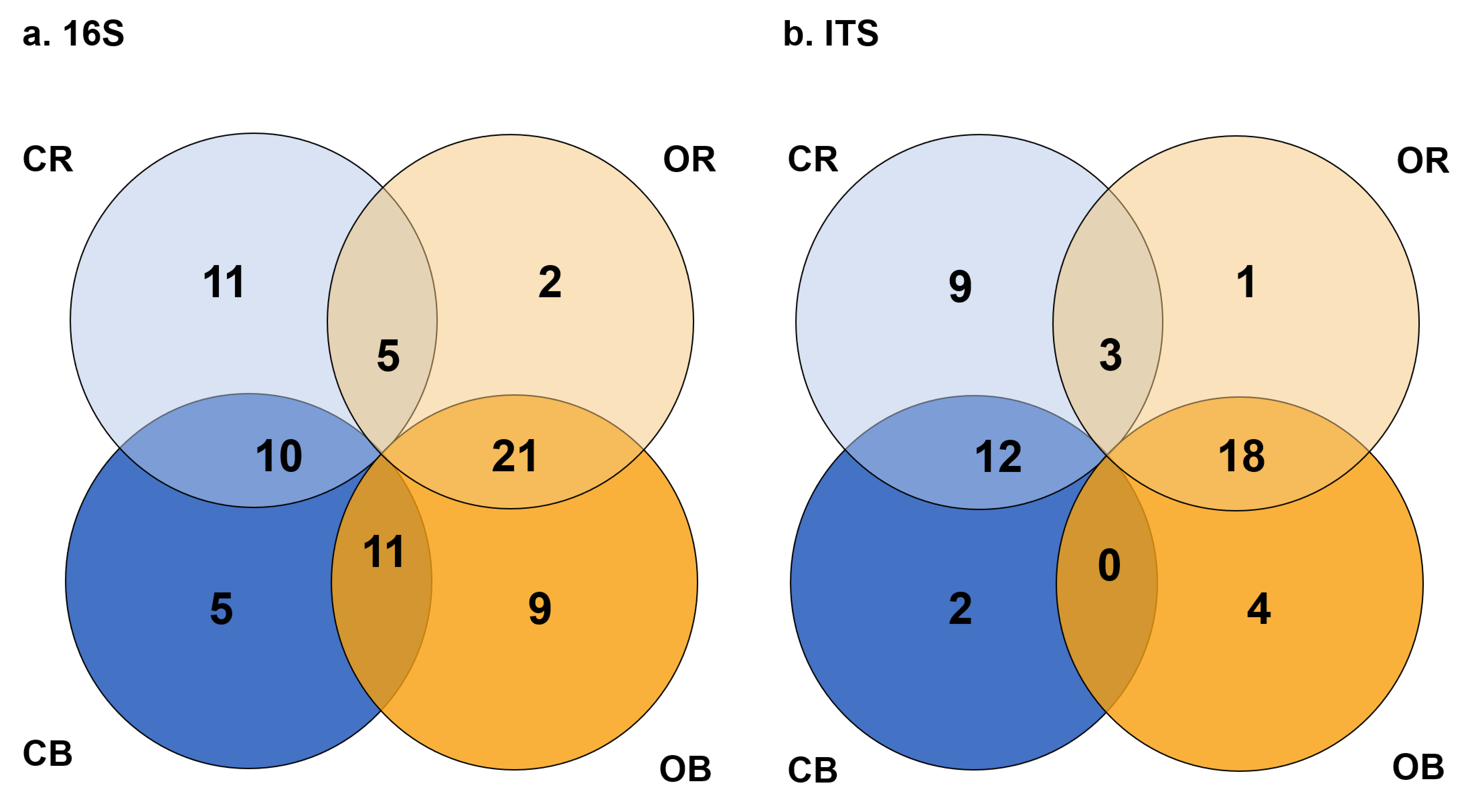

Supplement: Supplementary file 10 — Additional file 10: Figure S3. Indicator species analysis. A) Seventy-four bacterial and B) 49 fungal ASVs were identified as indicator species, occurring often in a given environment and rarely elsewhere. Far more ASVs were management-specific than rhizosphere-specific. Taxonomic information about the indicator ASVs can be found in Additional file 4: Table S3 and Additional file 5: Table S4. C = conventional, O = organic, B = bulk, R = rhizosphere. [file 40168_2019_756_MOESM10_ESM.tif]

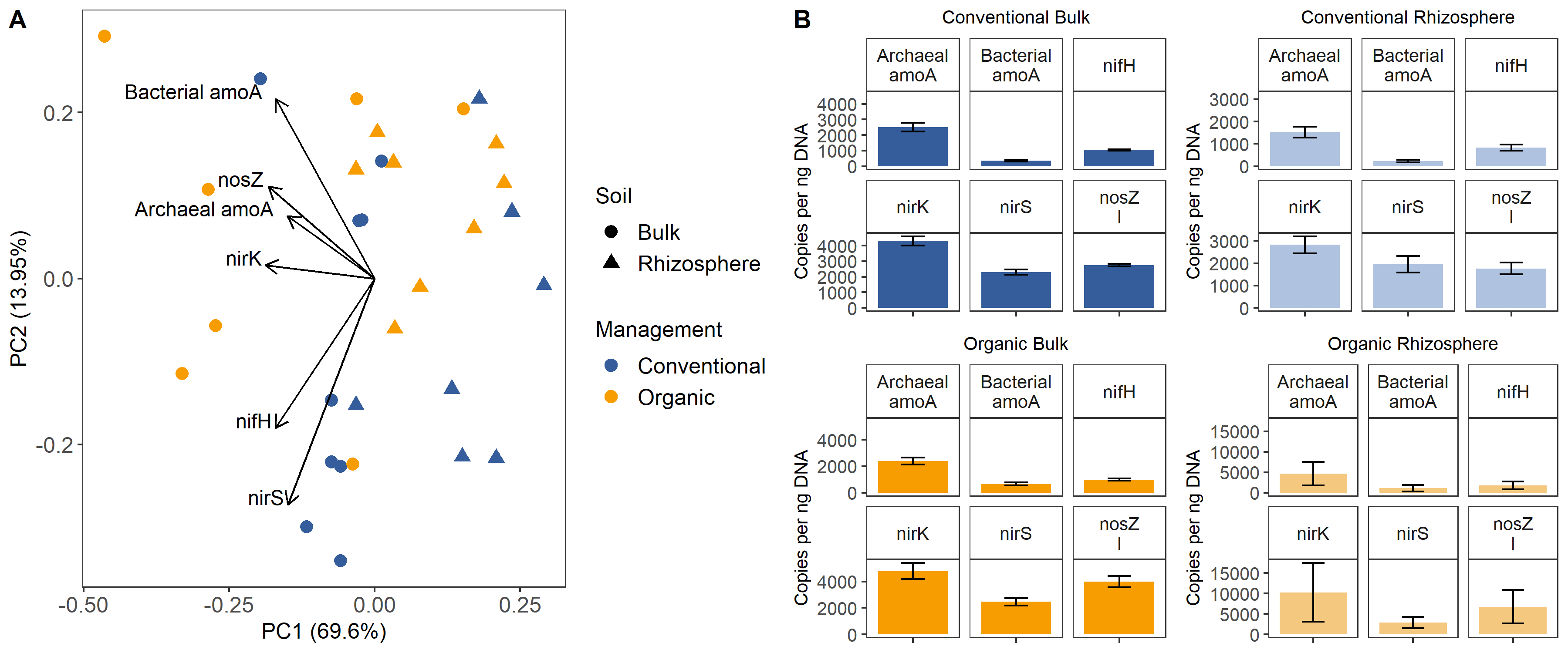

Supplement: Supplementary file 11 — Additional file 11: Figure S4. Multivariate analysis of N-cycling functional genes. A) Principal components analysis (PCA) revealed that samples separated primarily by soil compartment and secondarily by management system along the first principal component axis, which explained 69.6% of variation. B) Patterns of gene abundances for the N-cycling genes amoA (archaeal and bacterial), nifH, nirK, nirS, and nosZ were similar for all treatments, suggesting that system-specific bottlenecks in the N cycle were not observed. Accordingly, PERMANOVA revealed significant effects of soil compartment (p < 0.001) and management (p < 0.05) but not their interaction (p > 0.05). [file 40168_2019_756_MOESM11_ESM.tif]
